# Supplementary material for: Th17 cells target the metabolic miR‐142‐5p–succinate dehydrogenase subunit C/D (SDHC/SDHD) axis, promoting invasiveness and progression of cervical cancers
Source: Mol Oncol. 2023 Nov 16;18(9):2157–78. doi: 10.1002/1878-0261.13546 (PMC11467798; doi:10.1002/1878-0261.13546)

Supplementary Figure S1:
full unedited gels.

Full unedited gels for Figure 1B, **SiHa**

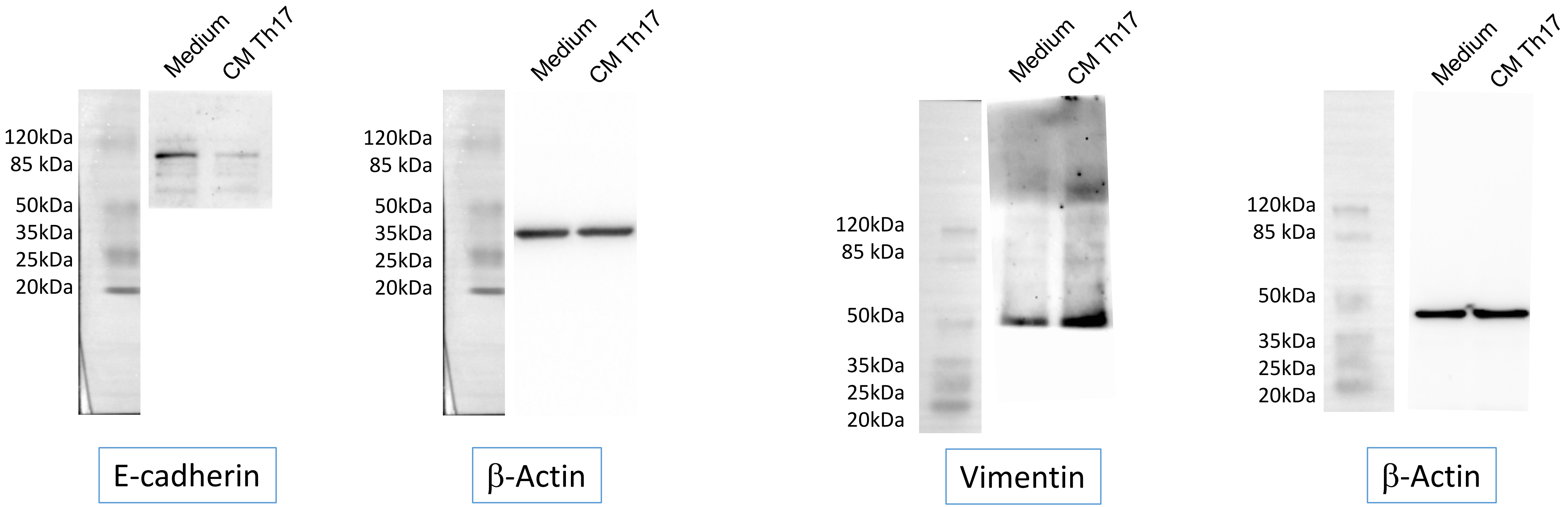

Supplementary Figure S1:
full unedited gels.

Full unedited gels for Figure 1B, **SiHa**

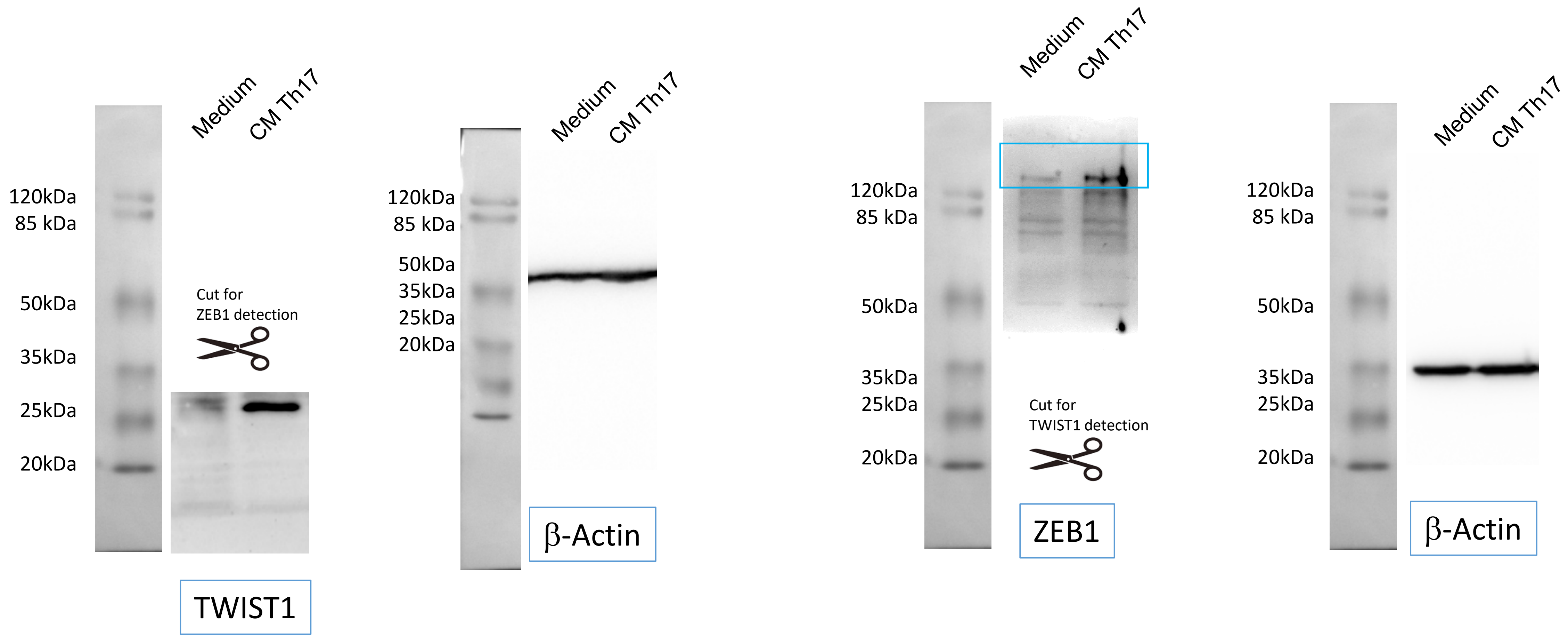

Supplementary Figure S1:
full unedited gels.

Full unedited gels for Figure 1B, **SW756**

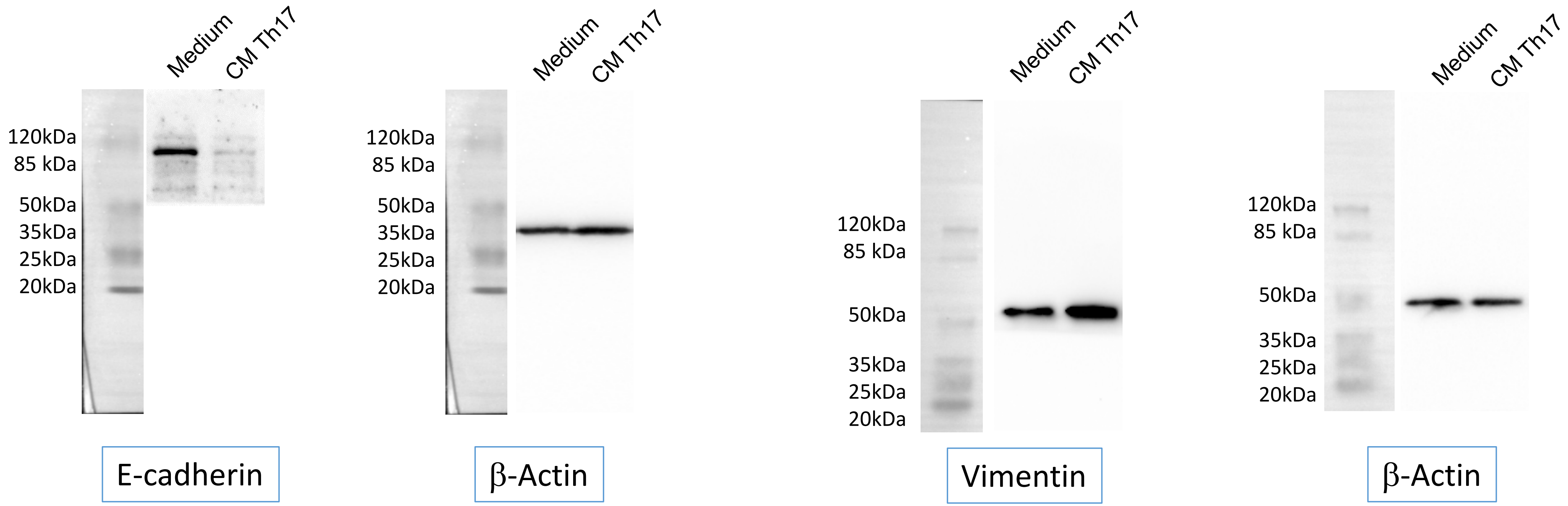

Supplementary Figure S1:
full unedited gels.

Full unedited gels for Figure 1B, **SW756**

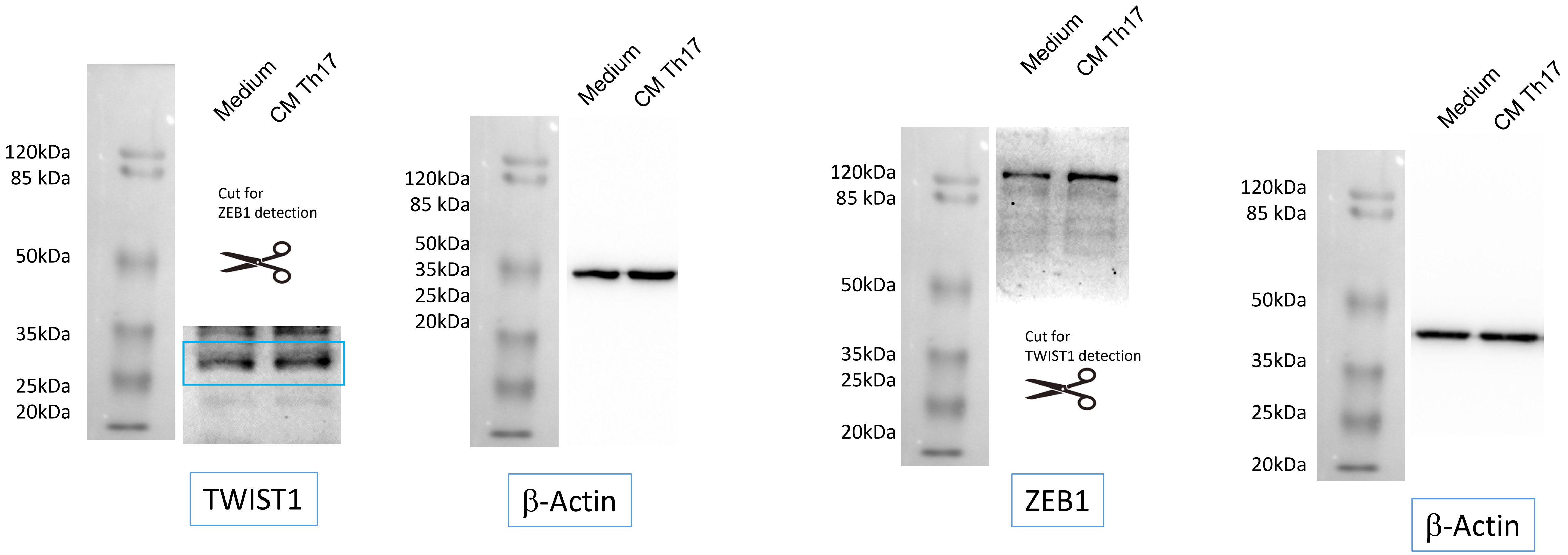

Supplementary Figure S1:
full unedited gels.

Full unedited gels for Figure 3C, **SiHa**

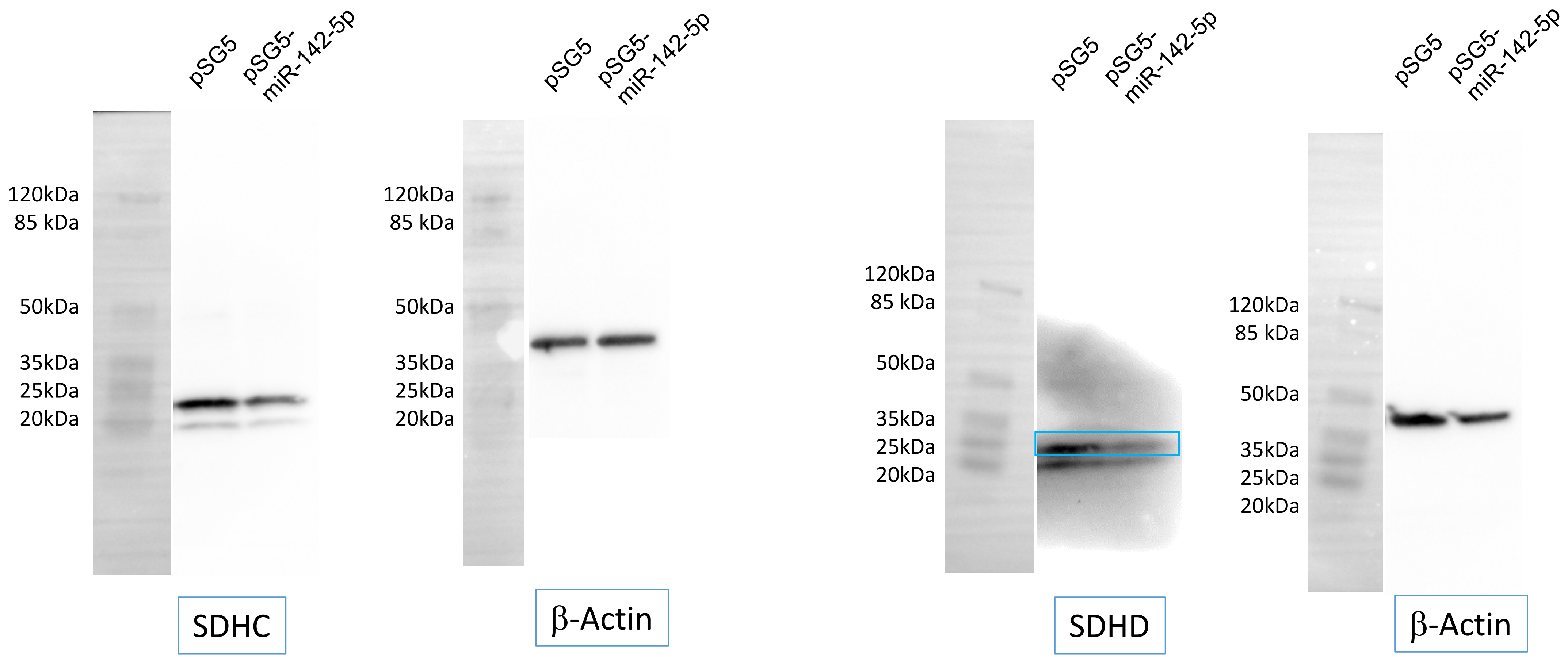

Supplementary Figure S1:
full unedited gels.

Full unedited gels for Figure 3C, **SW756**

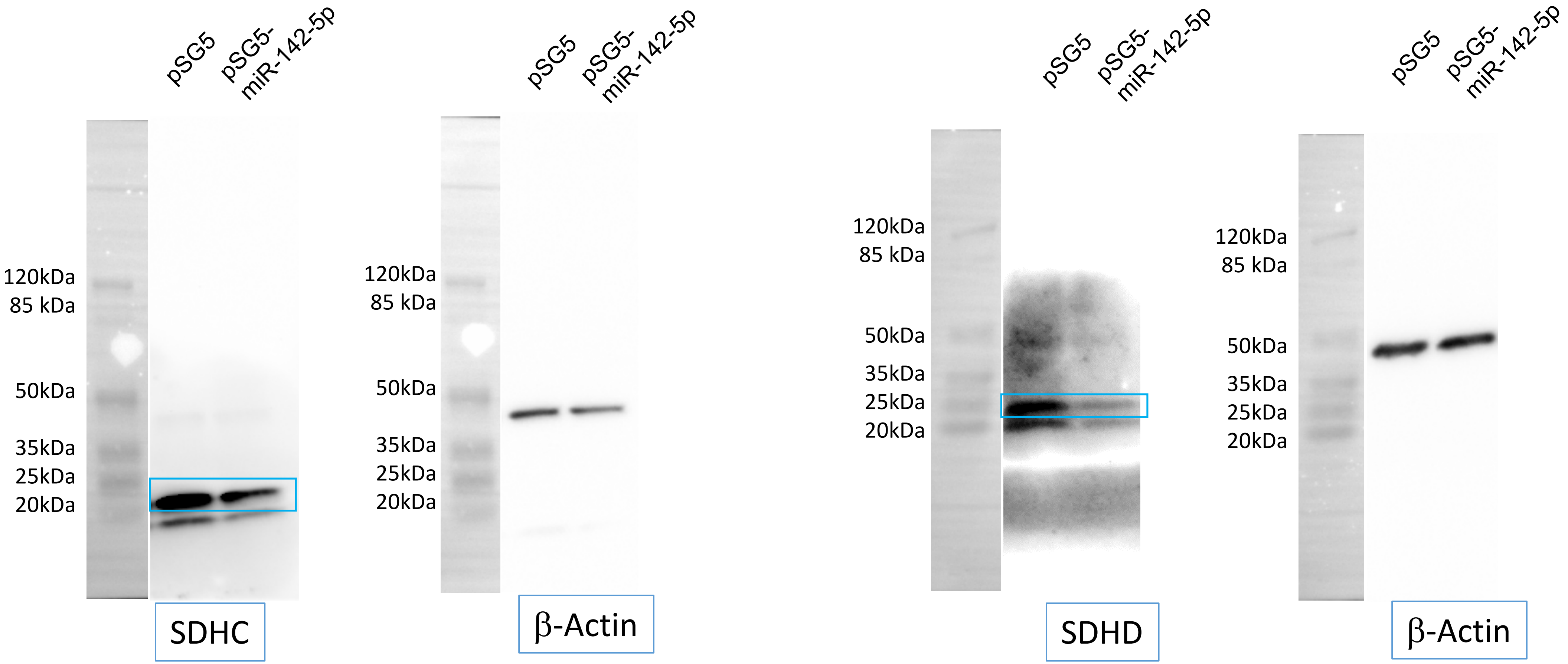

Supplementary Figure S1:
full unedited gels.

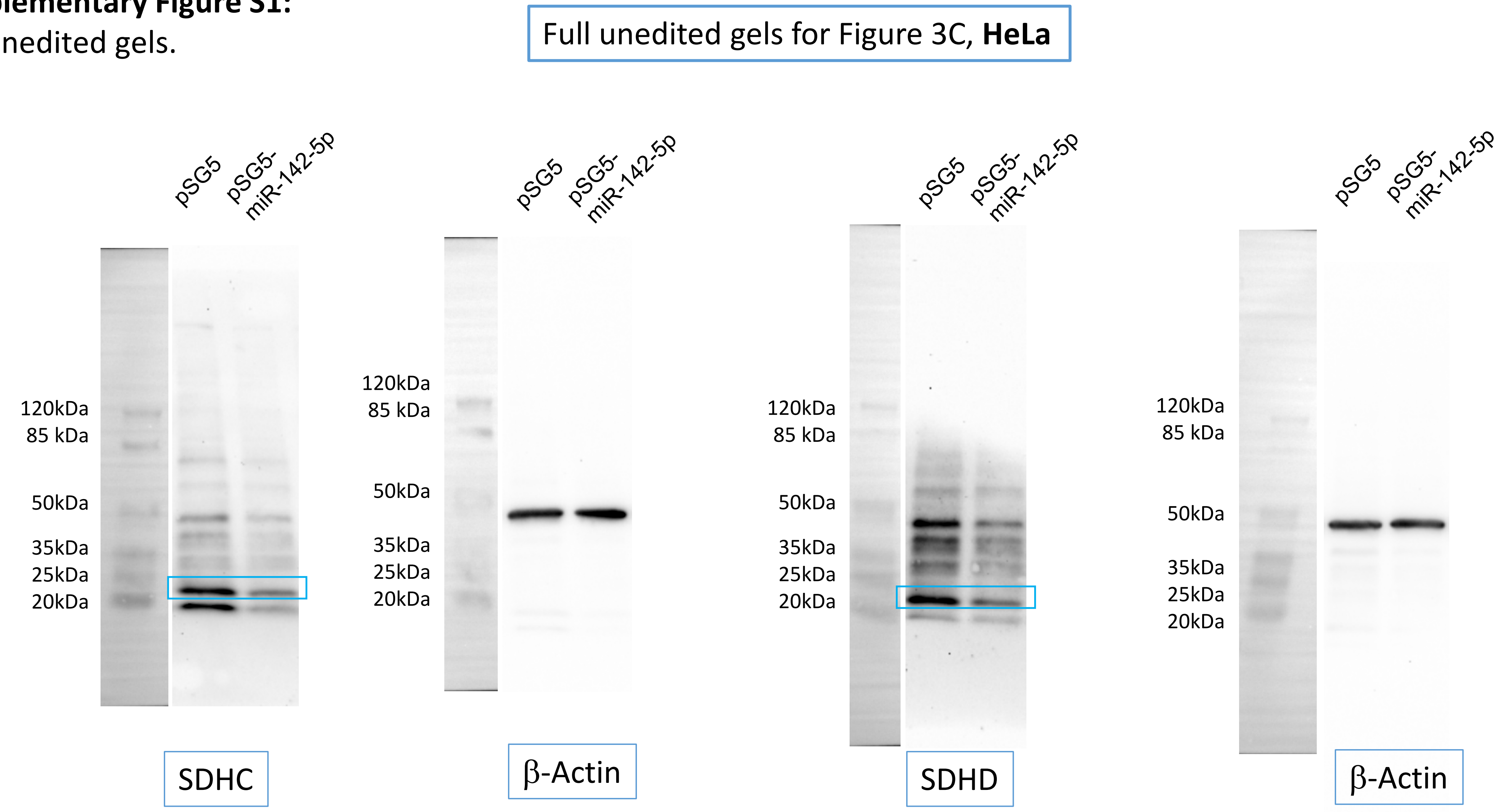

Supplementary Figure S1:
full unedited gels.

Full unedited gels for Figure 4E, **SiHa** (upper panel, SDHC)

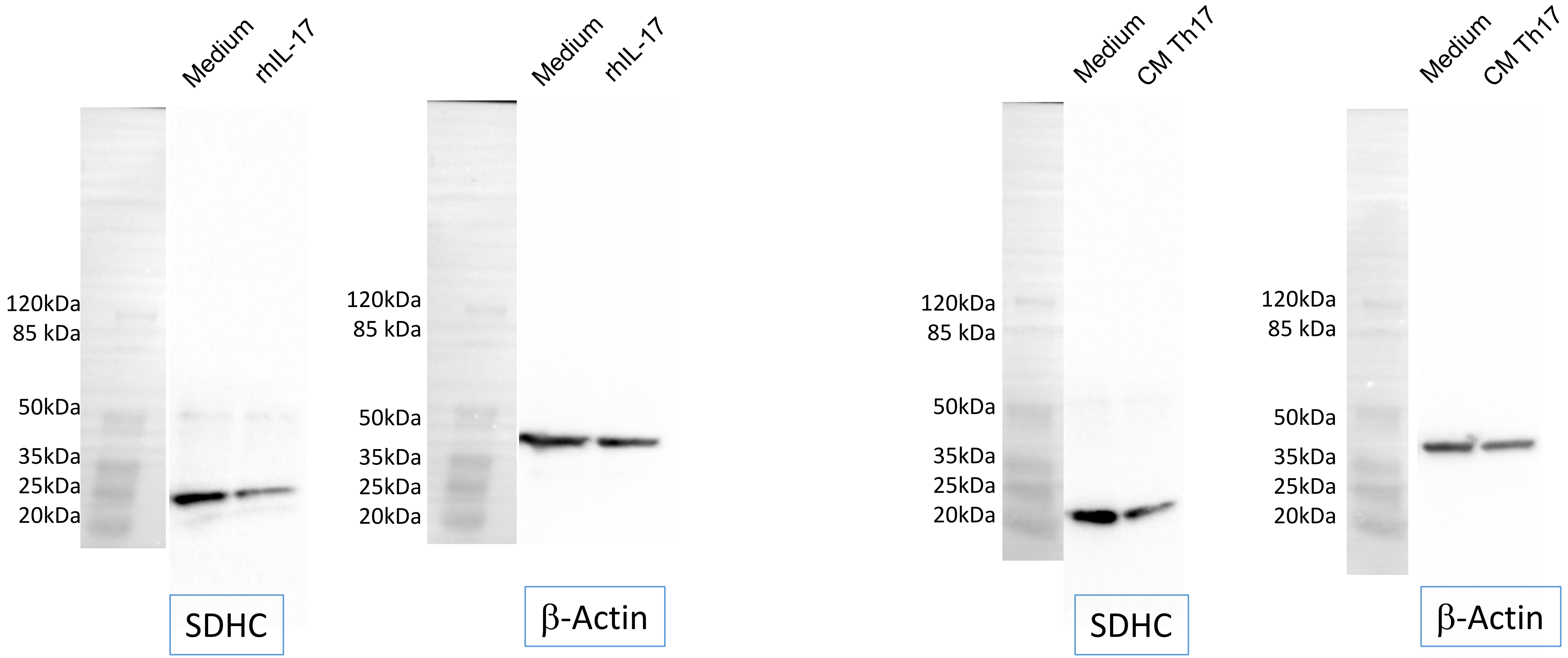

Supplementary Figure S1:
full unedited gels.

Full unedited gels for Figure 4E, **SiHa** (**lower panel, SDHD**)

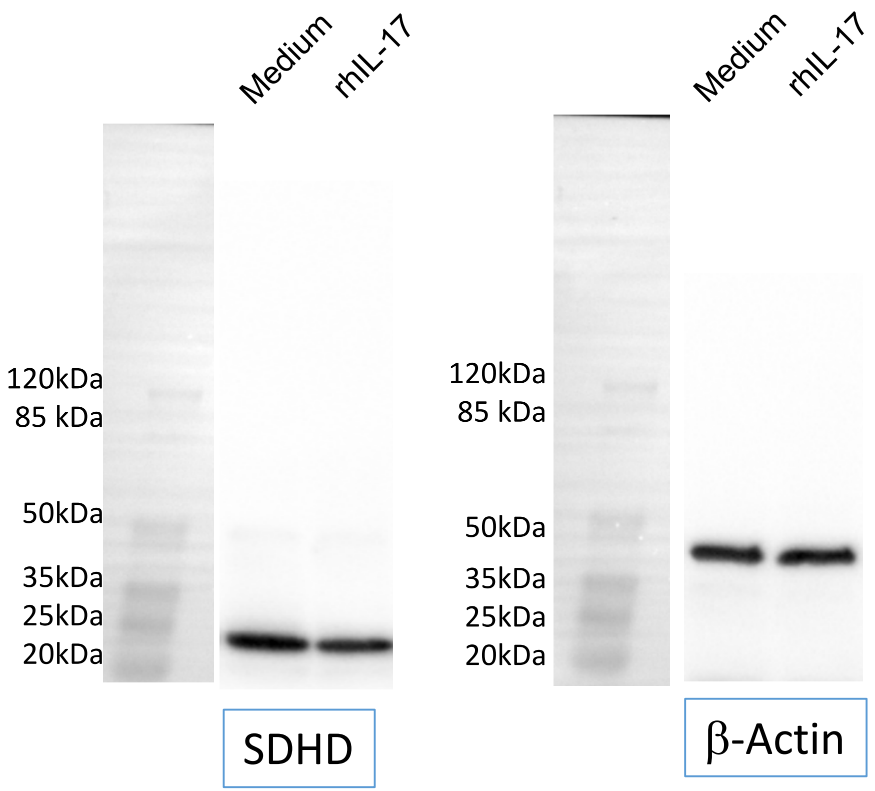

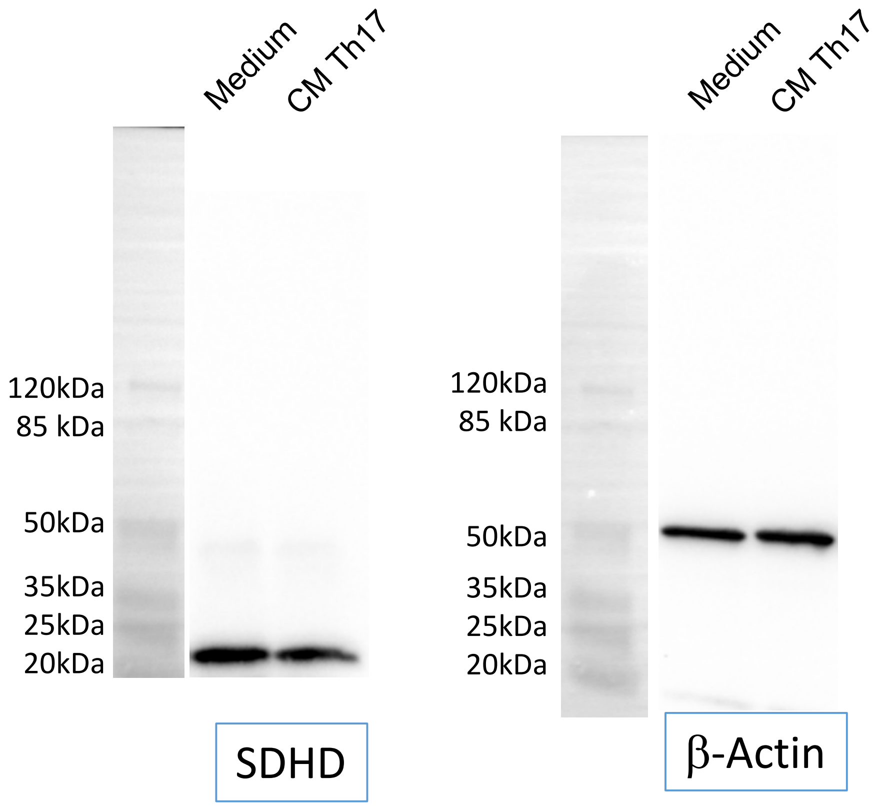

Supplementary Figure S1:
full unedited gels.

Full unedited gels for Figure 4E, **SW756** (**upper panel, SDHC**)

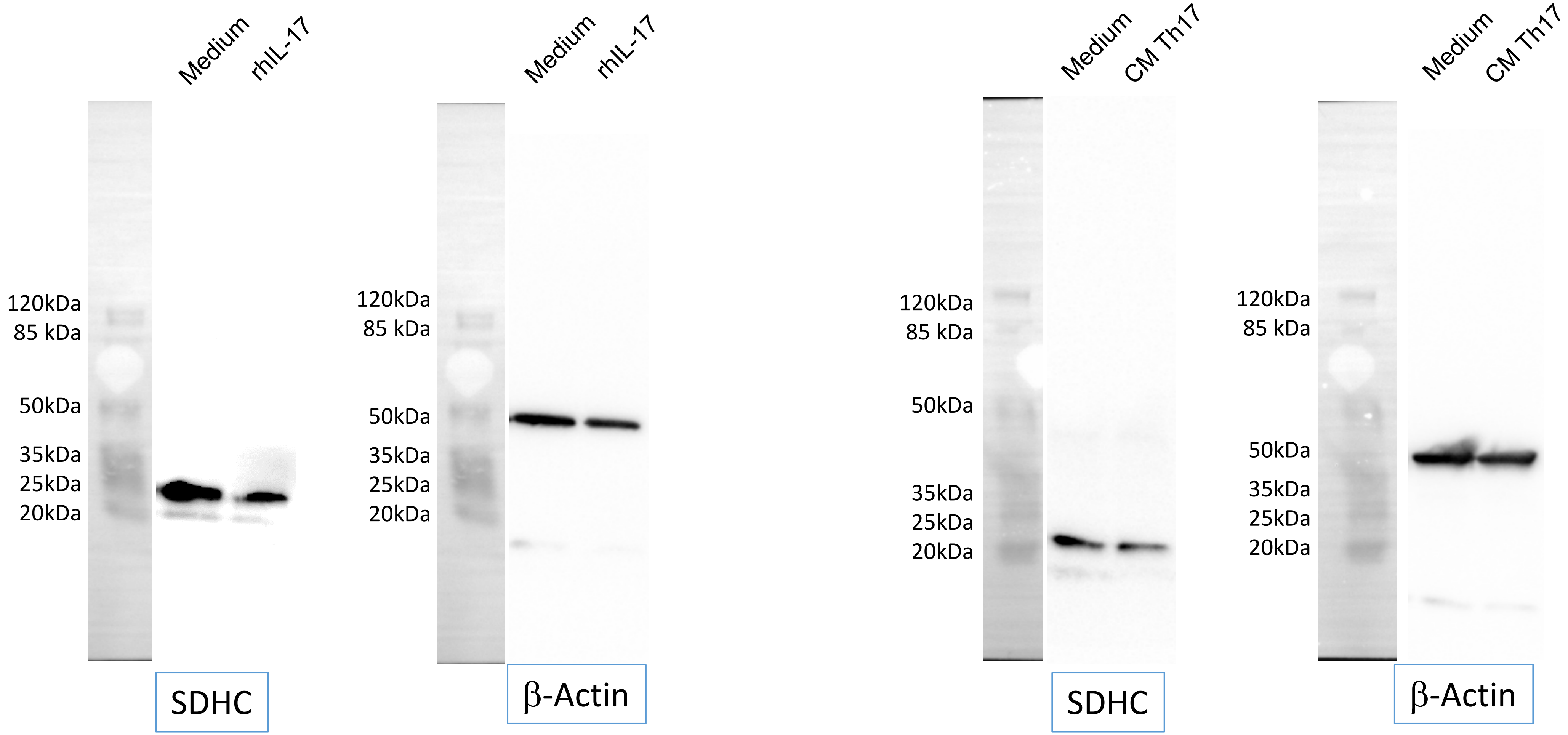

Supplementary Figure S1:
full unedited gels.

Full unedited gels for Figure 4E, **SW756** (lower panel, SDHD)

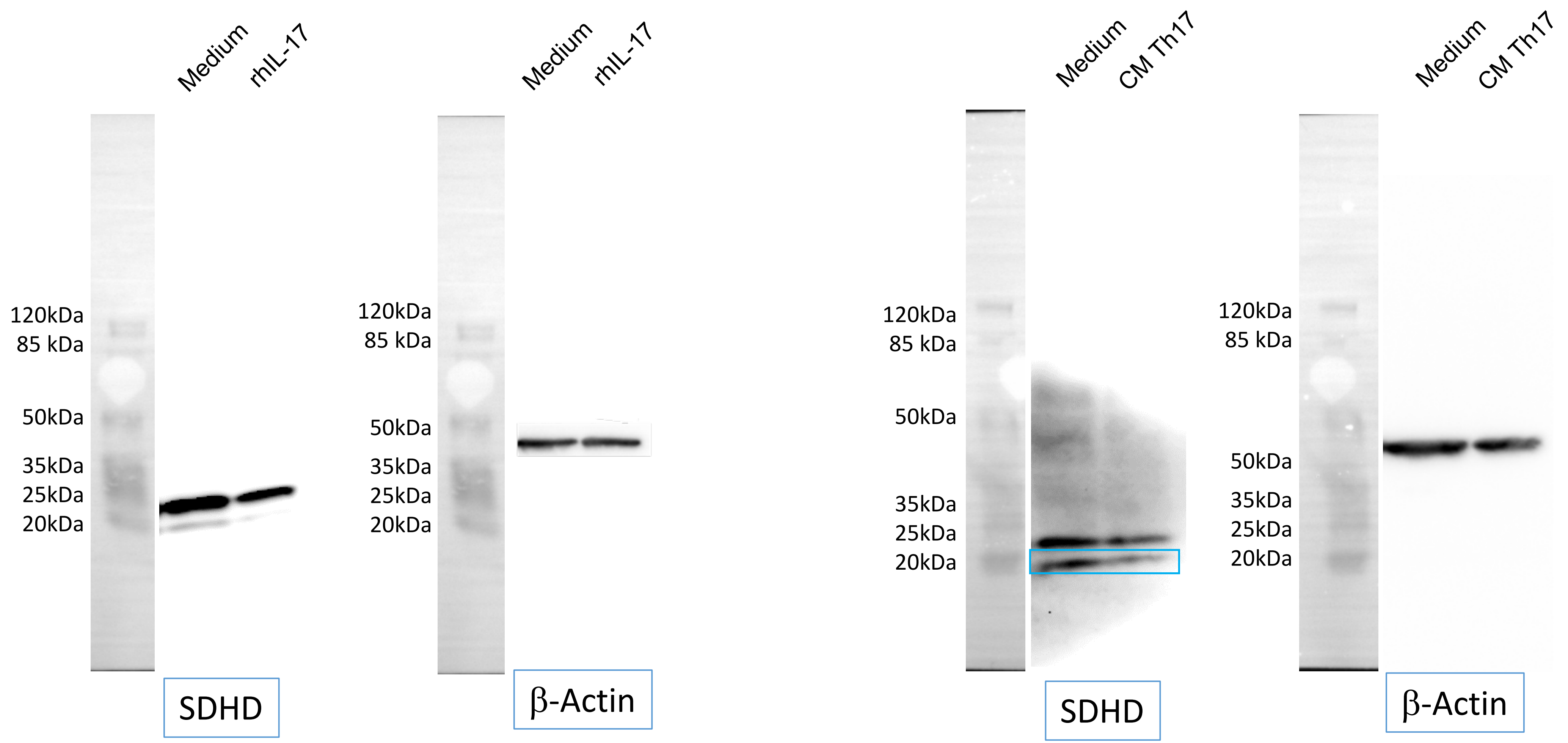

Supplementary Figure S1:
full unedited gels.

Full unedited gels for Figure 4E, **HeLa** (upper panel, SDHC)

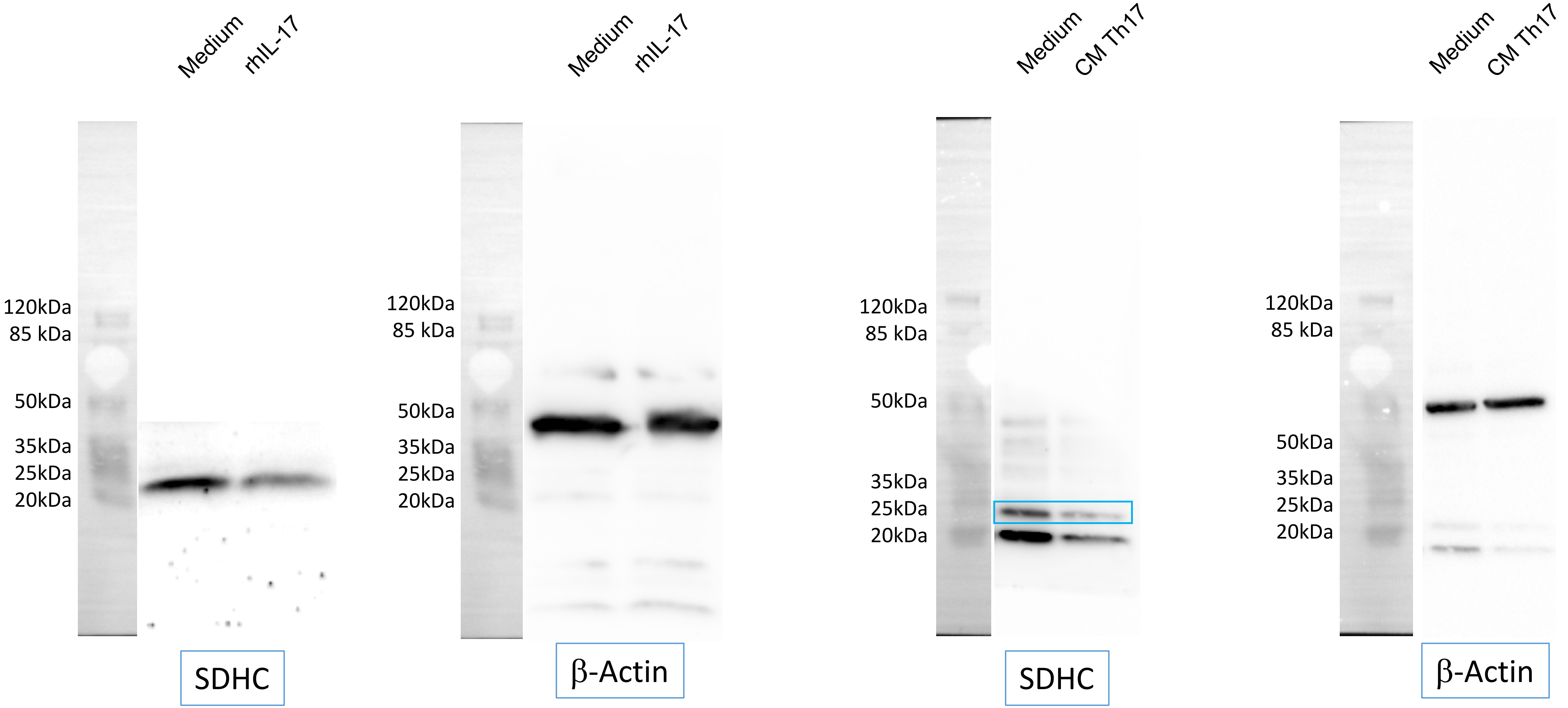

Supplementary Figure S1:
full unedited gels.

Full unedited gels for Figure 4E, **HeLa** (lower panel, SDHD)

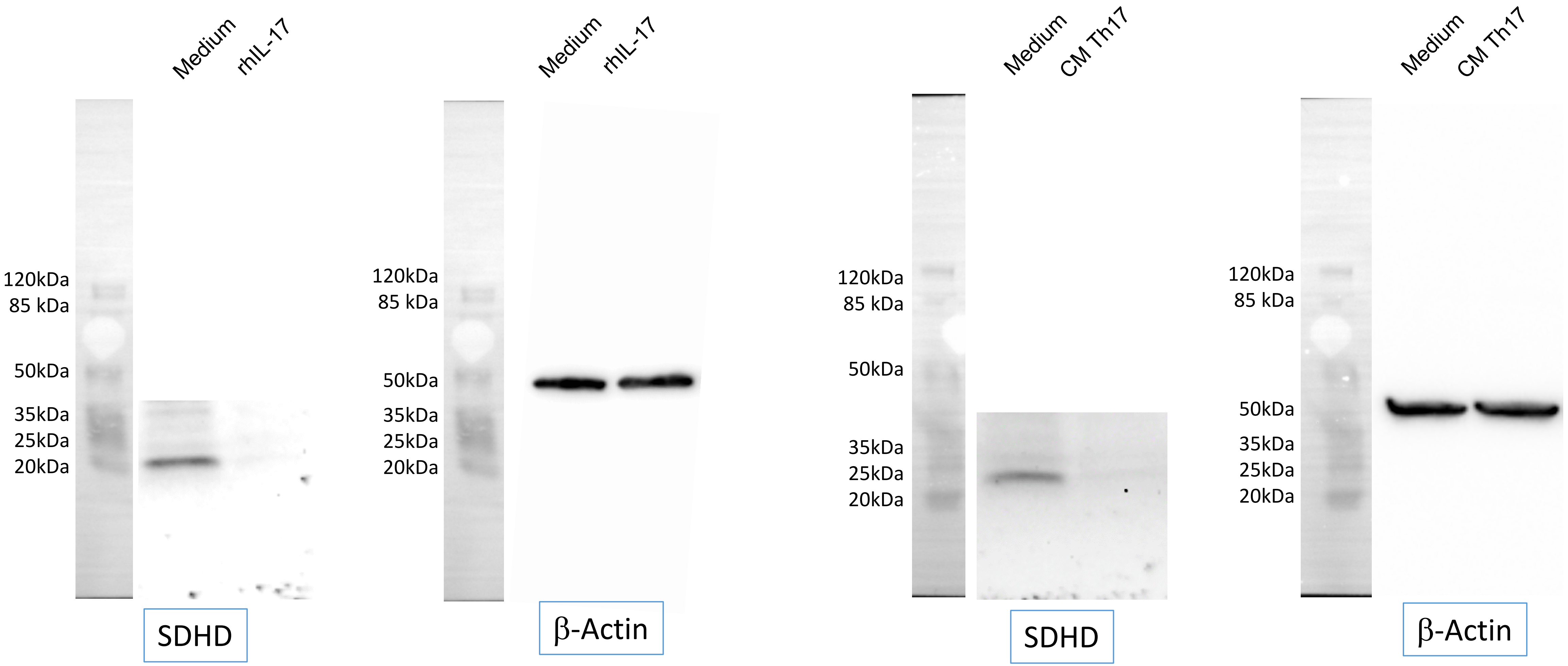

Supplementary Figure S1:
full unedited gels.

Full unedited gels for Figure 5A, **SiHa**

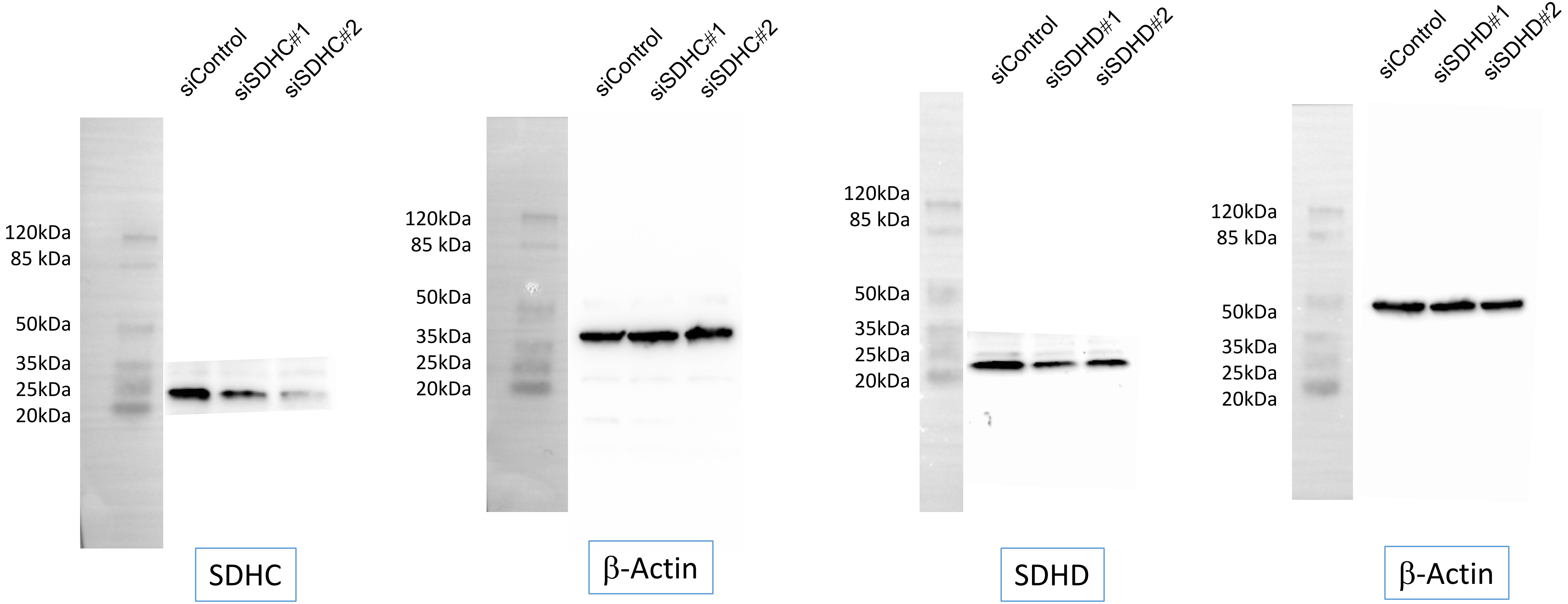

Supplementary Figure S1:
full unedited gels.

Full unedited gels for Figure 5A, **HeLa**

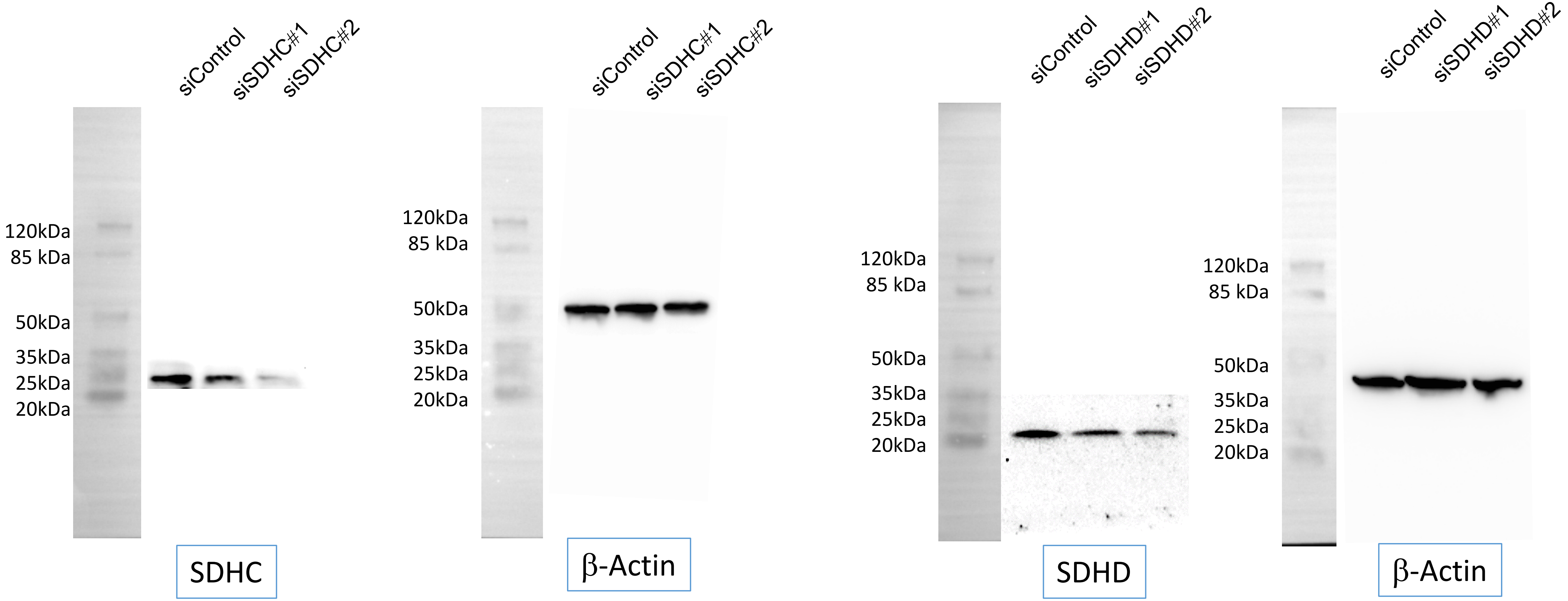

Supplementary Figure S1:
full unedited gels.

Full unedited gels for Figure 5A, **SiHa**

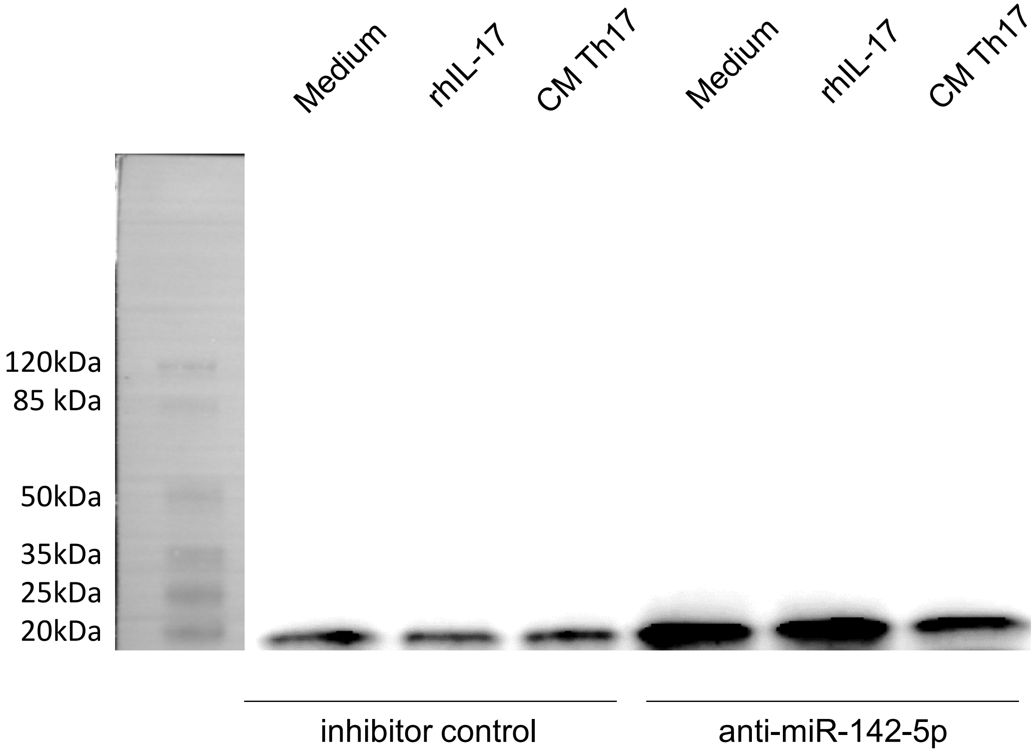

SDHC

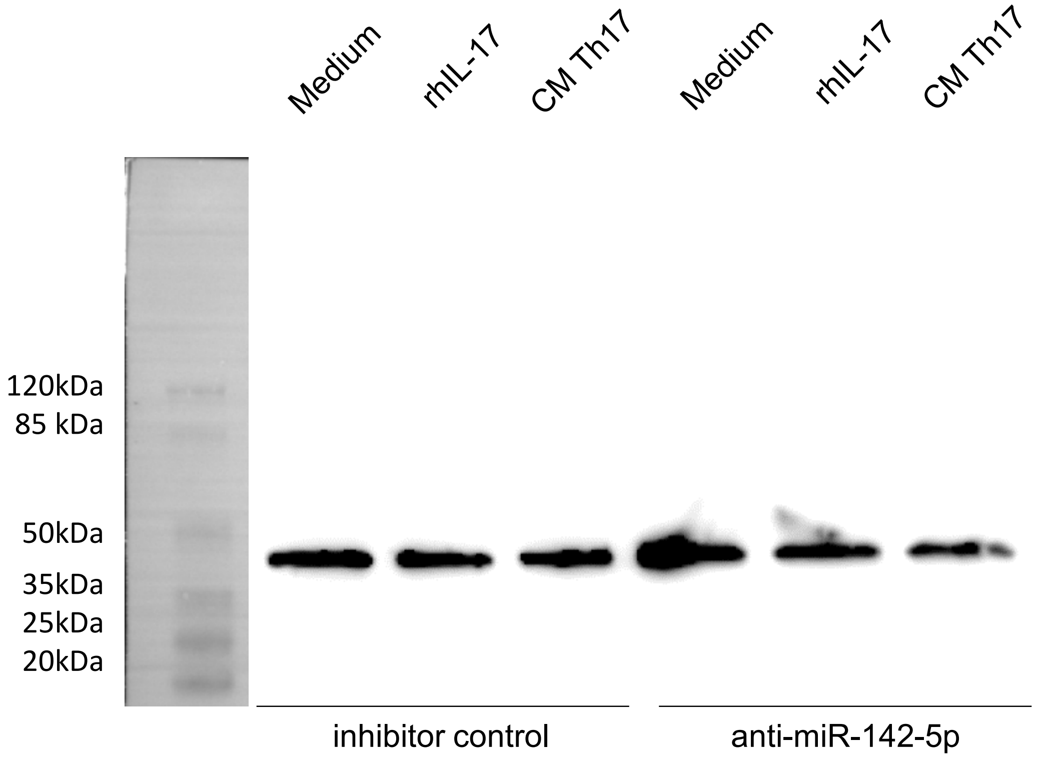

β-Actin

Supplementary Figure S1:
full unedited gels.

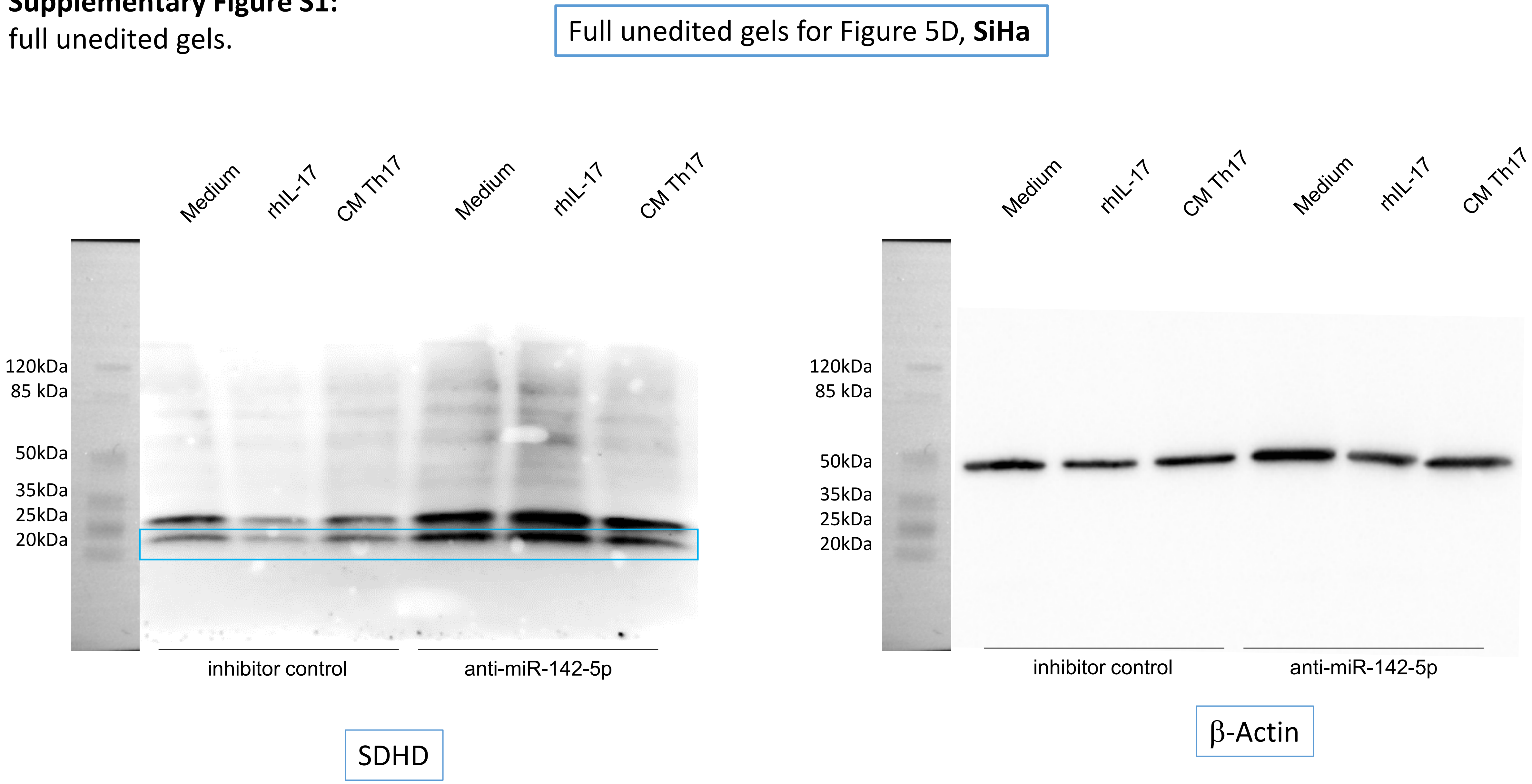

Supplementary Figure S1:
full unedited gels.

Full unedited gels for Supplementary Figure S3C, **SiHa**

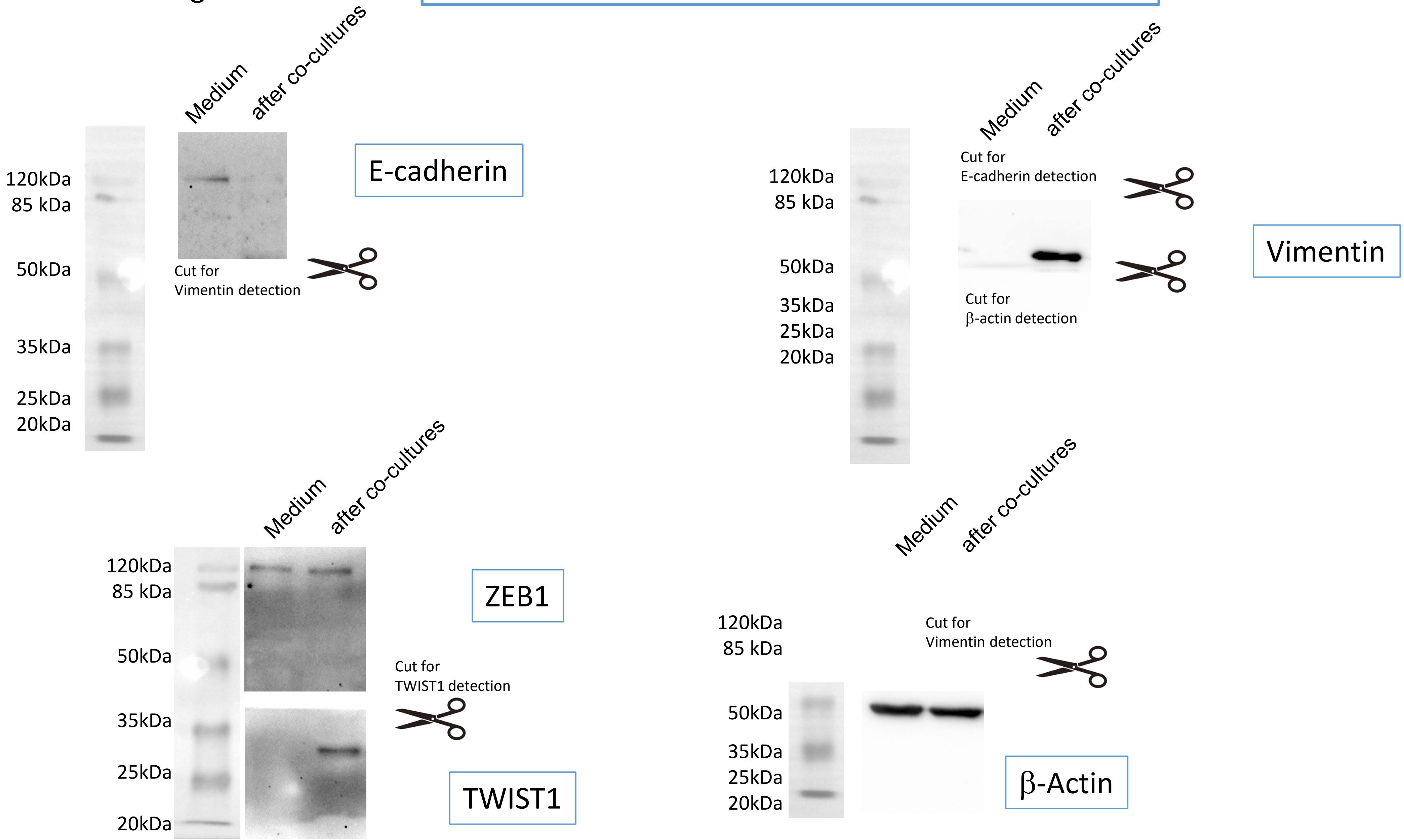

Supplementary Figure S1:
full unedited gels.

Full unedited gels for Supplementary Figure S3C, **SW756**

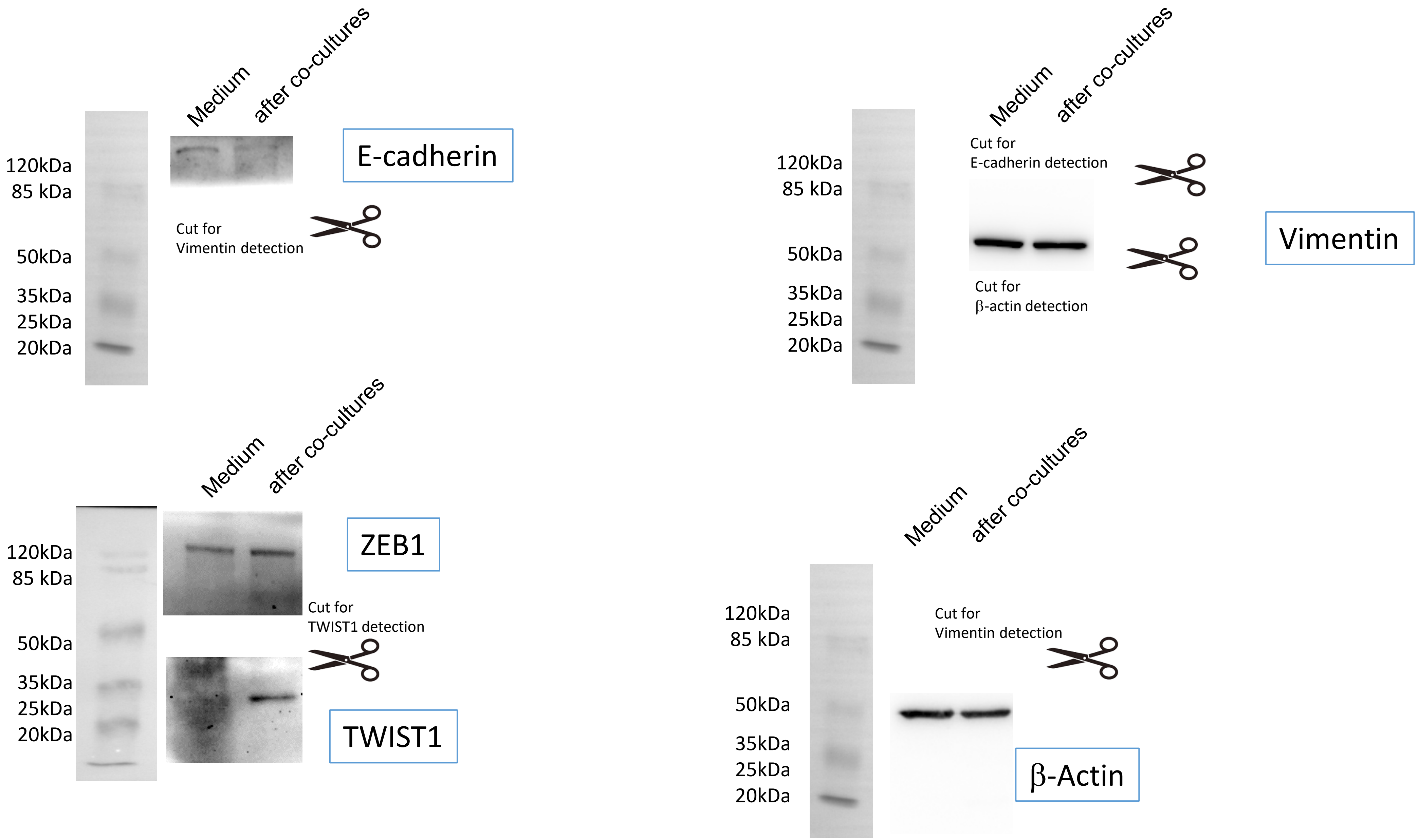

Supplementary Figure S1: full unedited gels.

Full unedited gels for Supplementary Figure S6C, **SiHa**

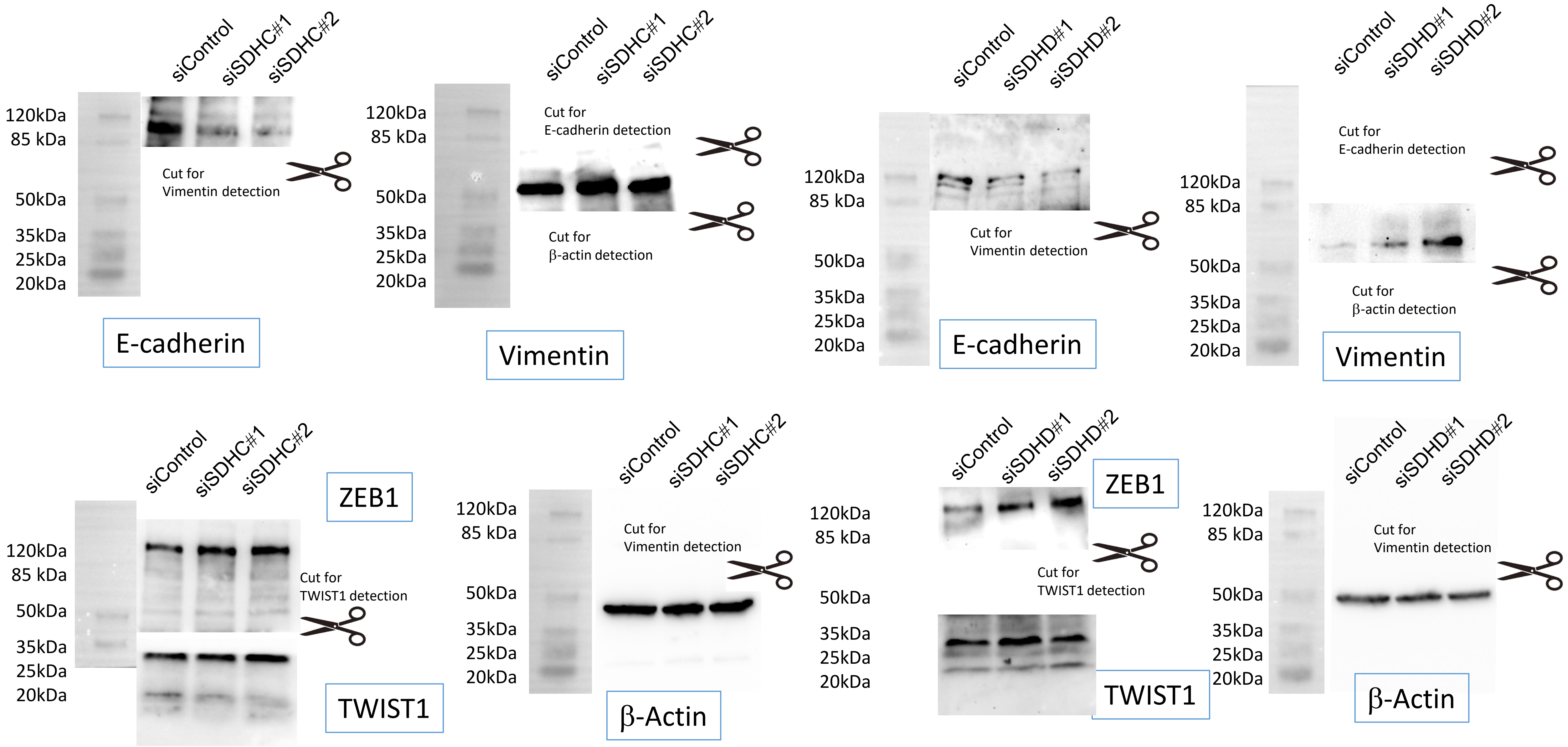

Supplementary Figure S1:
full unedited gels.

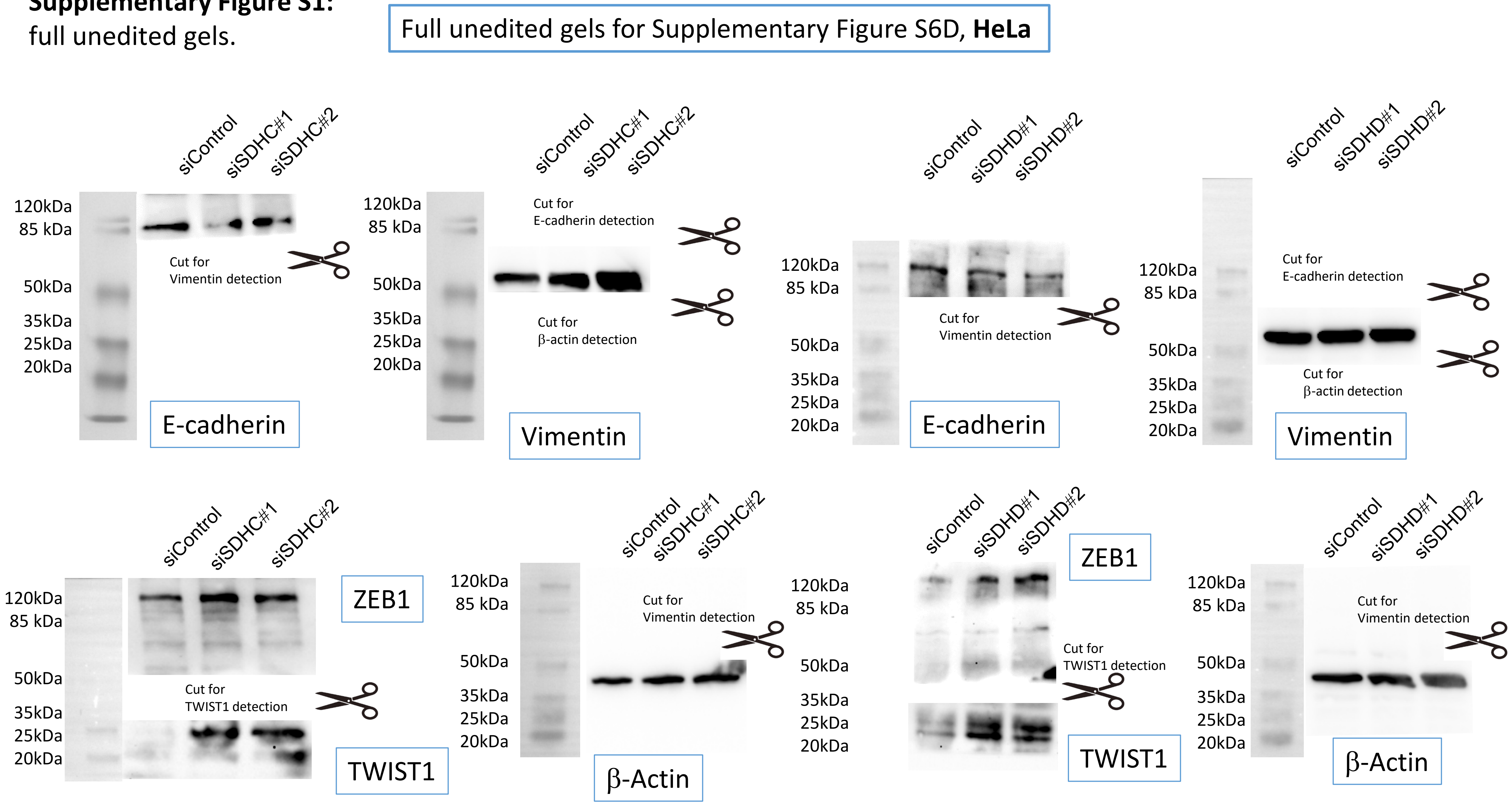

Supplement: Supplementary file 1 — Fig. S1. Full unedited gels. [file MOL2-18-2157-s010.pdf]
