# Supplementary figures and images for: Th17 cells target the metabolic miR‐142‐5p–succinate dehydrogenase subunit C/D (SDHC/SDHD) axis, promoting invasiveness and progression of cervical cancers
Source: Mol Oncol. 2023 Nov 16;18(9):2157–78. doi: 10.1002/1878-0261.13546 (PMC11467798; doi:10.1002/1878-0261.13546)

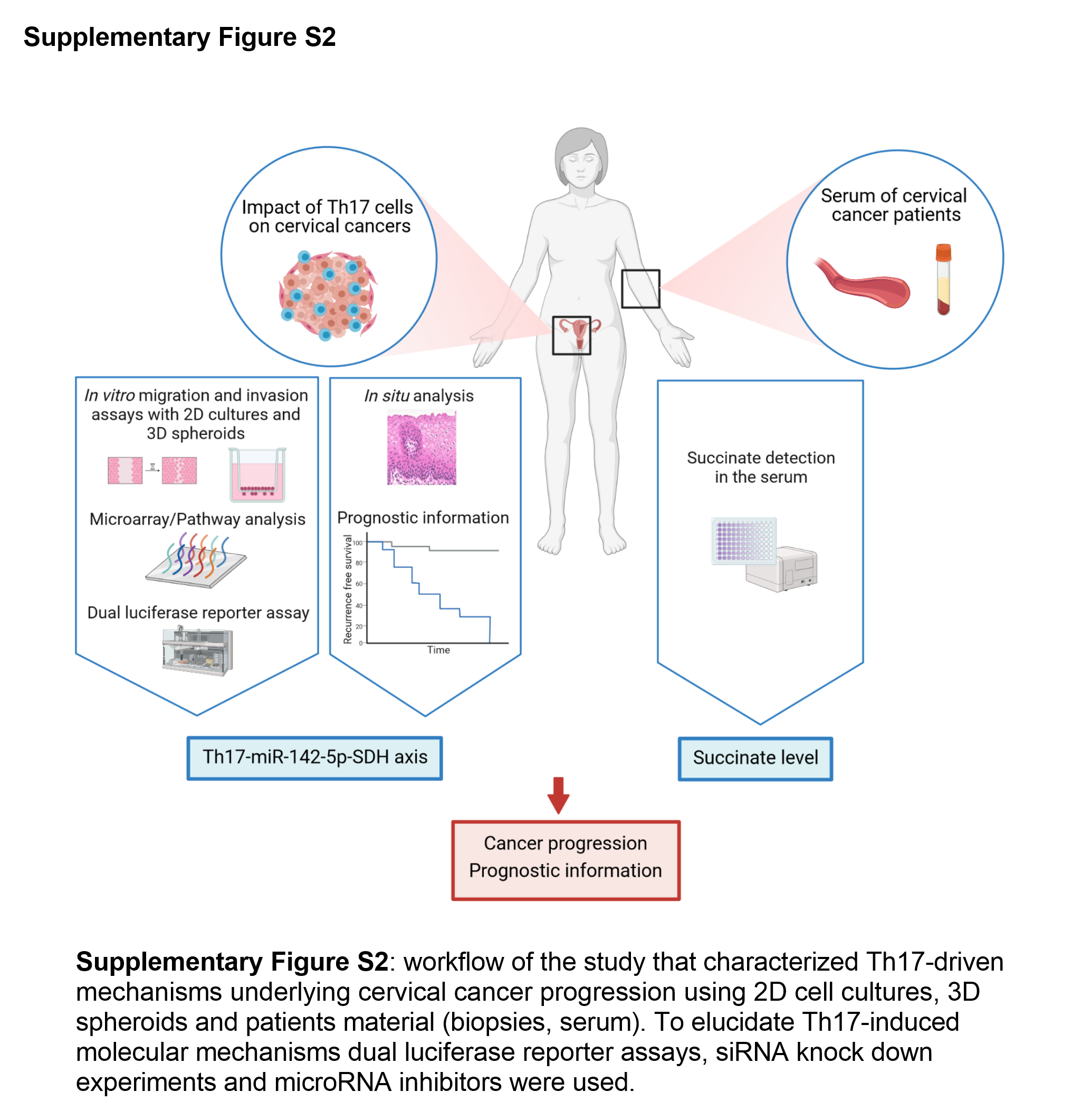

Supplement: Supplementary file 2 — Fig. S2. Workflow of the study. [file MOL2-18-2157-s004.tif]

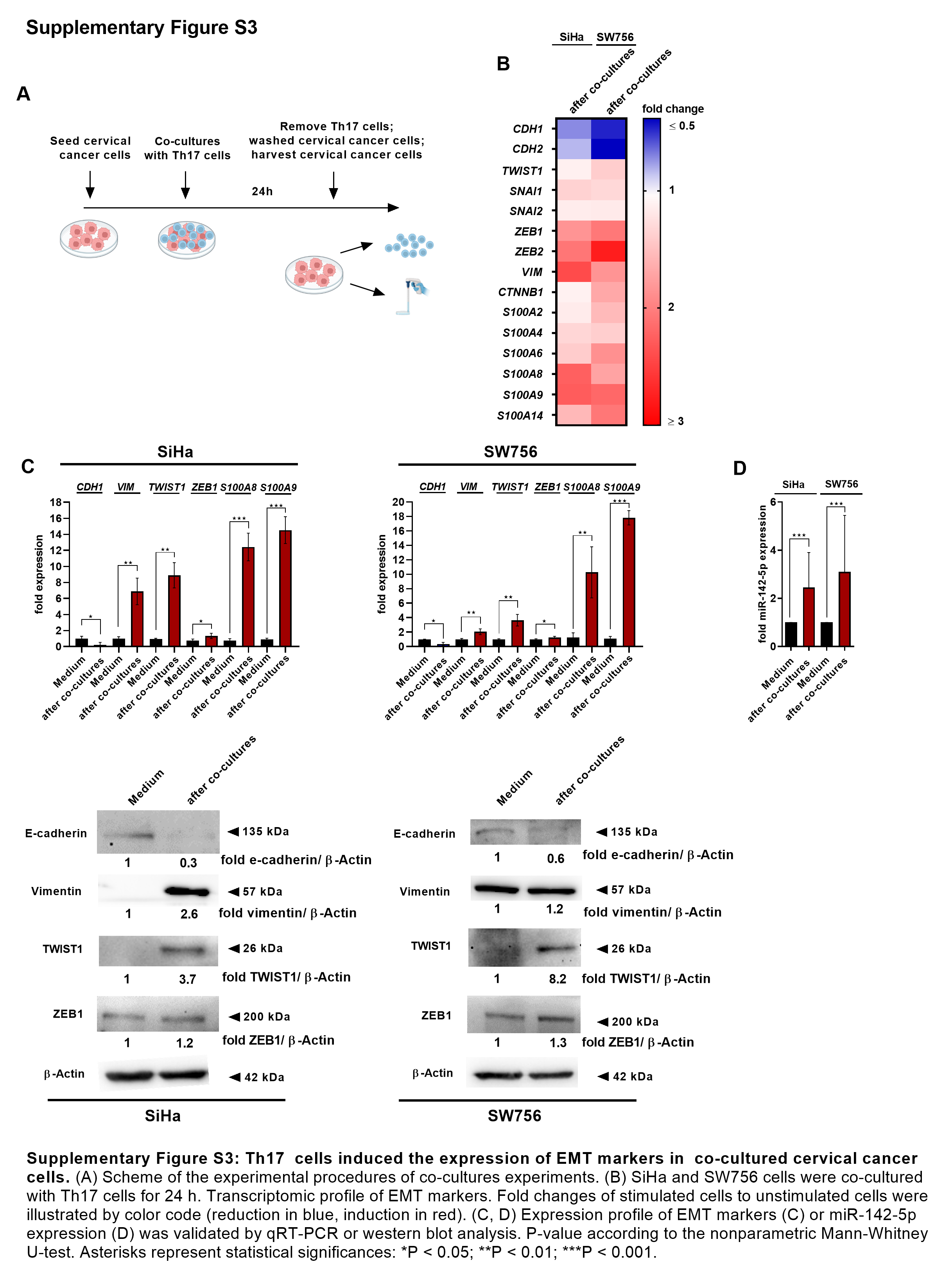

Supplement: Supplementary file 3 — Fig. S3. h17 cells induced the expression of EMT markers in co‐cultured cervical cancer cells. [file MOL2-18-2157-s001.tif]

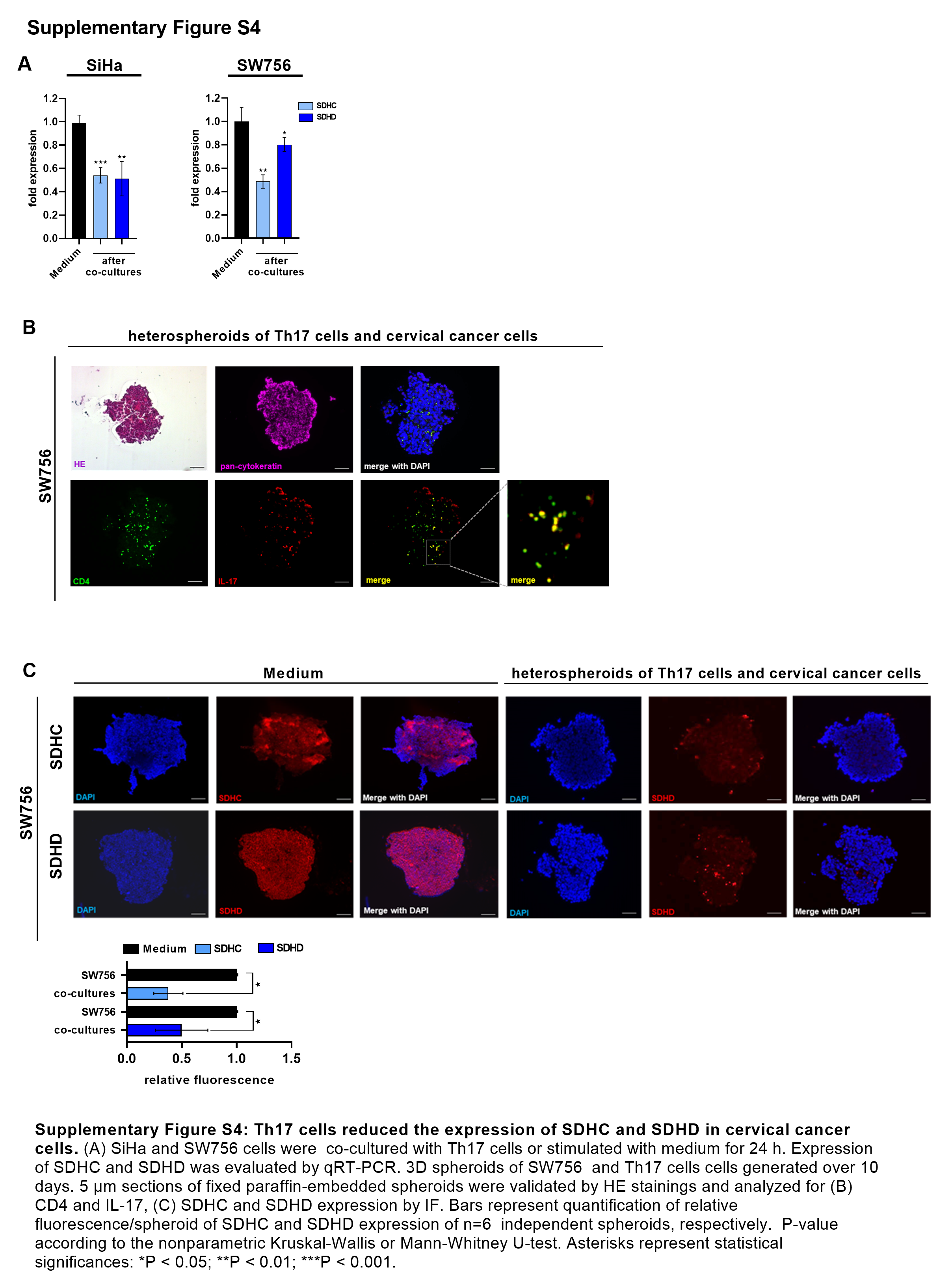

Supplement: Supplementary file 4 — Fig. S4. Th17 cells reduced the expression of SDHC and SDHD in cervical cancer cells. [file MOL2-18-2157-s006.tif]

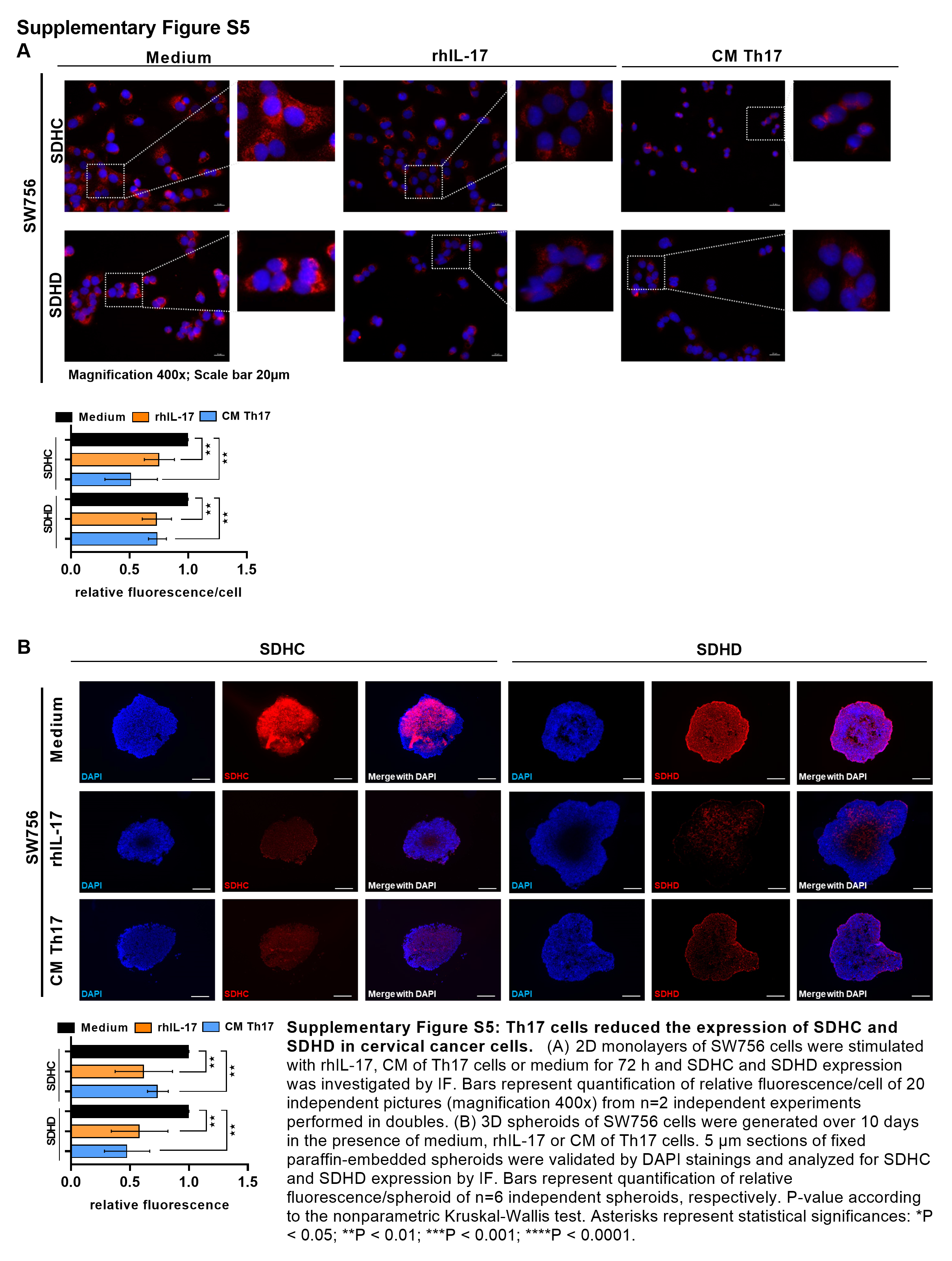

Supplement: Supplementary file 5 — Fig. S5. Th17 cells reduced the expression of SDHC and SDHD in cervical cancer cells. [file MOL2-18-2157-s009.tif]

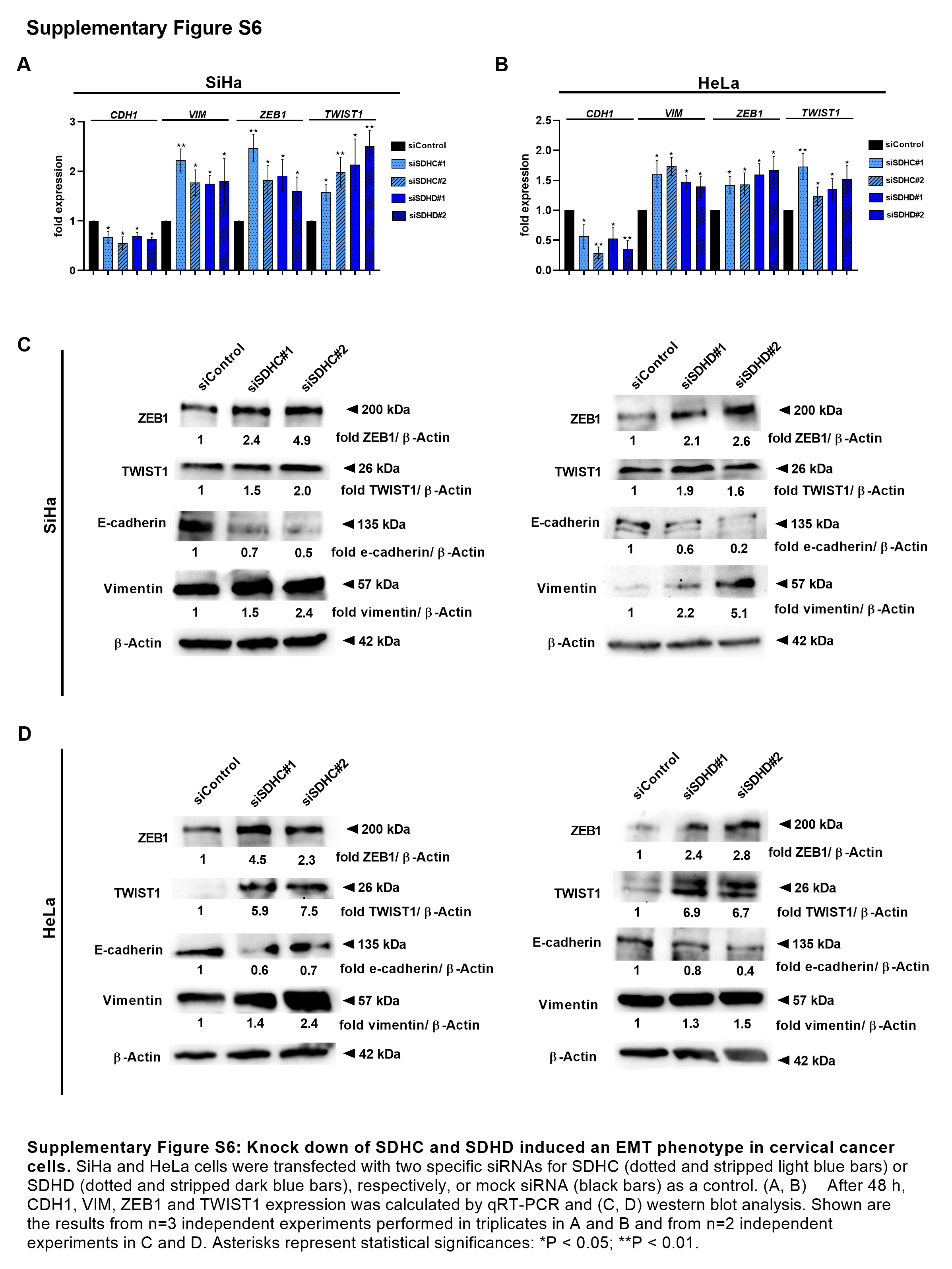

Supplement: Supplementary file 6 — Fig. S6. Knock down of SDHC and SDHD induced an EMT phenotype in cervical cancer cells. [file MOL2-18-2157-s008.tif]

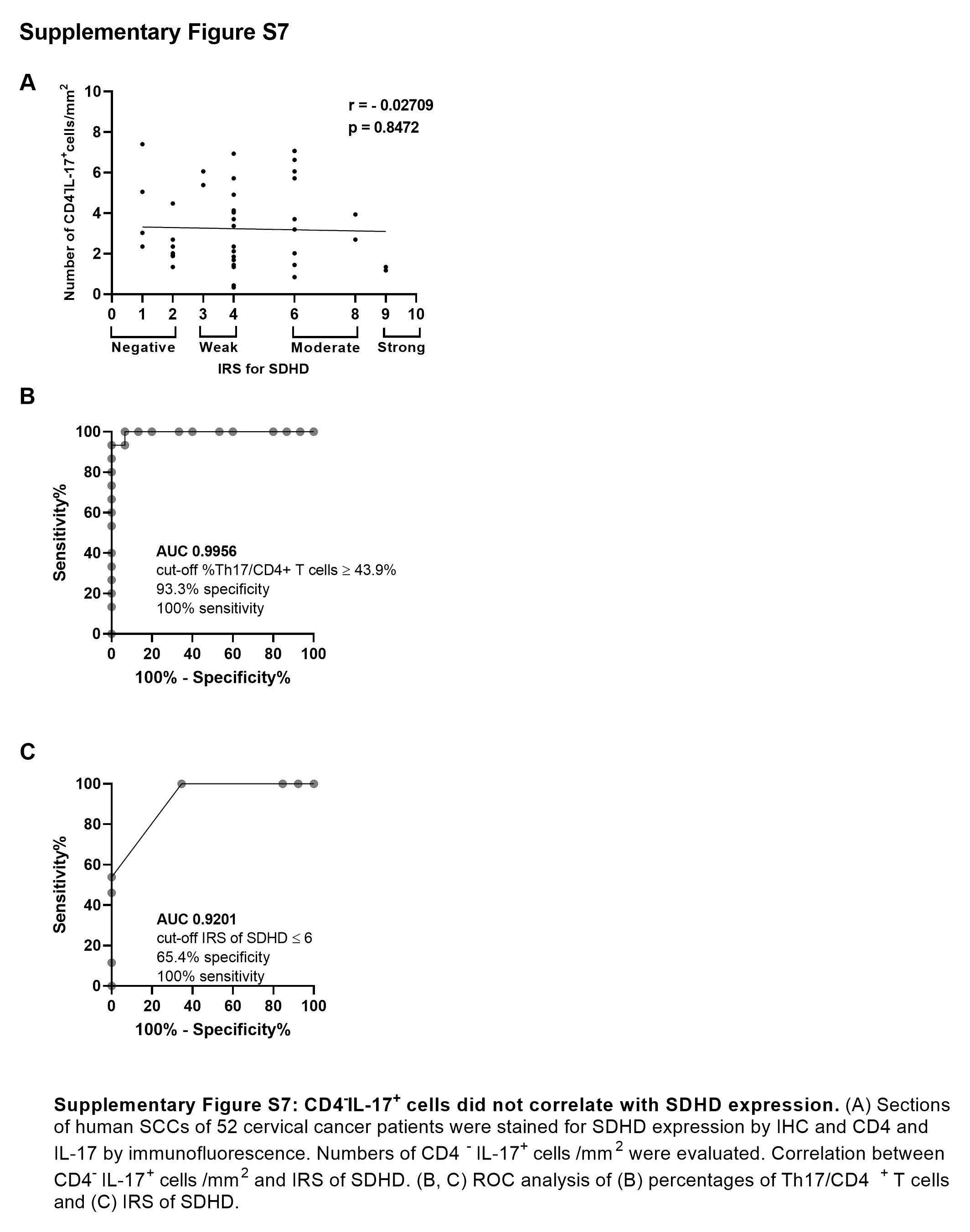

Supplement: Supplementary file 7 — Fig. S7. CD4−IL‐17+ cells did not correlate with SDHD expression. [file MOL2-18-2157-s005.tif]
