# Supplementary material for: Th17 cells target the metabolic miR‐142‐5p–succinate dehydrogenase subunit C/D (SDHC/SDHD) axis, promoting invasiveness and progression of cervical cancers
Source: Mol Oncol. 2023 Nov 16;18(9):2157–78. doi: 10.1002/1878-0261.13546 (PMC11467798; doi:10.1002/1878-0261.13546)
Supplement: Supplementary file 8 — Table S1. List of used materials. [file MOL2-18-2157-s007.pdf]

Supplementary Table S1:

List of used materials.

**ANTIBODIES**			
**Antigen/Protein**	**Dilution/ concentration**	**Vendor**	**Identifier**
rabbit anti IL-17 polyclonal antibody	1:1000 for IF; Fig. 6	Abcam	#ab79056 RRID:AB_1603584
mouse anti CD4 monoclonal antibody, clone 4B12,	1:500 for IF; Fig. 6	Leica Biosystems,	#CD4-368-L-CE-H RRID:AB_563560
mouse anti human CD4 PE monoclonal antibody, clone RPA-T4	5µl pro test	BD Biosciences	#555347 RRID:AB_395752
anti-CCR6-Alexa Fluor647 (11A9)	10µl per test	BD Biosciences, Heidelberg, Germany	#560466 RRID:AB_1645402
rabbit anti SDHC	1:200 for WB; Fig. 1, 3, 4, 5 and Supplementary Figure S5	Fisher Scientific	#PA5-49522 RRID: AB_2634976
rabbit anti SDHD	1:200 for WB; Fig. 1, 3, 4, 5 and Supplementary Figure S6	Fisher Scientific	#PA5-71645 RRID: AB_2717499
mouse anti-TOMM22/TOM22 antibody	1:200 for IF; Fig. 4	Abcam	#ab57523 RRID:AB_945897
mouse anti e-cadherin	1:1000 for WB; Fig. 1, S5	Cell Signaling	#14472 RRID: AB_2728770
rabbit anti vimentin	1:1000 for WB; Fig. 1, S5	Cell Signaling	#5741 RRID: AB_10695459
rabbit anti TWIST1	1:1000 for WB; Fig. 1, S5	Cell Signaling	#69366 AB_2891135
Rabbit anti ZEB1	1:1000 for WB; Fig. 1, S5	Cell Signaling	#70512 AB_2935802
mouse anti β-actin, clone AC-15	1:5000 for WB; Fig. 3, 4, 5	Sigma-Aldrich,	#A5441 RRID:AB_476744
Human IL-17/IL-17A Antibody	1µg/ml for neutralization; Fig. 2	R&D Systems	#AF-317-SP RRID:AB_354463
normal goat IgG control	1µg/ml for neutralization; Fig. 2	R&D Systems	#AB-108-C RRID: AB_354267
Goat anti-rabbit IgG (H+L) Highly Cross-Adsorbed Secondary Antibody, Alexa Fluor™ 546	1:200 for IF; Figure 4 and Supplementary Figure S4, 5	Invitrogen	#A-11035 RRID: AB_2534093
Goat anti-mouse IgG (H+L) Highly Cross-Adsorbed Goat anti-Mouse, Alexa Fluor™ 488	1:200 for IF; Figure 4 and Supplementary Figure S4	Invitrogen	#A-11029 RRID:AB_2534088

**CHEMICALS, PEPTIDES, AND RECOMBINANT PROTEINS**		
Human IL-17, research grade	Miltenyi Biotec	#130-094-625
Human IL-6	PROSPEC	#CYT-213
Human TGF-β1, premium grade	Miltenyi Biotec	#130-095-067
Human IL-1β	PEPROTECH	#200-01B
Human IL-23, research grade	Miltenyi Biotec	#130-095-757
RPMI-1640 medium	Sigma-Aldrich	#R8758
Dulbecco’s Modified Eagle’s Medium - high glucose	Sigma-Aldrich	#D5796
Dulbecco’s Phosphate Buffered Saline	Sigma-Aldrich	#D8537
Fetal Bovine Serum	Sigma-Aldrich	#F7524
Fetal Bovine Serum, qualified	Gibco	#10270-106
Sodium pyruvate solution	Sigma-Aldrich	#S8636
Amersham™ Protran® Western blotting membranes, nitrocellulose	GE Healthcare Life Science	#10600002
4-15% Mini-PROTEAN® TGX™ Precast Protein Gels	BioRad	#4561084
QIAzol Lysis Reagent	QIAGEN	#79306
Opti-MEM™, GlutaMAX™ I	Thermo Fisher	#51985034
Lipofectamine™ RNAiMAX	Thermo Fisher	#13778150
Pancoll human	PAN biotech	#P04-601000

Phorbol 12-myristate 13-acetate (PMA)	Sigma-Aldrich	#P8139
Ionomycin	Sigma-Aldrich	#I0634
Brefeldin A	Sigma-Aldrich	#B6542
Matrigel® Growth Factor Reduced (GFR) Basement Membrane Matrix	Corning Costar Corp	#354230
Paraformaldehyde	Sigma-Aldrich	#P6148
**COMMERCIAL ASSAYS**		
Maxima Reverse Transcriptase	ThermoFisher Scientific	#EP0742
Fast Start Taq DNA Polymerase dNTPack	Roche	#4738357001
miRNeasy Mini KIT	Qiagen	#217084
miRCURY LNA RT Kit	Qiagen	#339340
miScript RT II Kit	Qiagen	Product leaked
miRCURY LNA SYBR Green PCR Kit	Qiagen	#339345
Dual-Luciferase® Reporter Assay System	Promega	#E1960
ImmPRESS® HRP Horse Anti-Rabbit IgG Polymer Detection Kit, Peroxidase	Vector Laboratories	#MP-7401
Invitrogen™ Molecular Probes™ TSA™ Kit 2, with HRP-Goat Anti-Mouse IgG and Alexa Fluor™ 488 Tyramide	Life Technology	#T20912
Invitrogen™ Molecular Probes™ TSA™ Kit 13, with HRP-Goat Anti-Rabbit IgG and Alexa Fluor™ 546 Tyramide	Life Technology	#T20923
Naive $CD4^+$ T Cell Isolation Kit II , human	Miltenyi Biotec	#130-094-131
T Cell Activation/Expansion Kit, human	Miltenyi Biotec	#130-091-441
IL-17 Secretion Assay – Cell Enrichment and Detection Kit (PE), human	Miltenyi Biotec	#130-094-542
SuperSignal West Dura Substrate	Thermo Fisher	#34076
Human IL-17 DuoSet ELISA	R&D Systems	#DY317
DuoSet ELISA Ancillary Reagent Kit 2	R&D Systems	#DY008
Succinate Colorimetric Assay Kit	Sigma-Aldrich	#MAK184
Venor®GeM Classic	Minerva Biolabs	#11-1025
**EXPERIMENTAL MODELS: CELL LINES**		
Human HPV-16 positive cervical cancer cell line SiHa	ATCC	CVCL_0032
Human HPV-18 positive cervical cancer cell line HeLa	ATCC	CVCL_0030
Human HPV-18 positive cervical cancer cell line SW756	ATCC	CVCL_1727
**OLIGONUCLEOTIDES**		
ON-TARGETplus Non-targeting siRNA #2; targeting sequence: UGGUUUACAUGUUGUGUGA	Horizon Discovery	D-001810-02
ON-TARGETplus siRNA SDHC #05, targeting sequence: GCACUGGUAUUGCUUUGAG	Horizon Discovery	J-011385-05
ON-TARGETplus siRNA SDHC #08, targeting sequence: AGAUAAAGAGGGCUAGUUA	Horizon Discovery	J-011385-08
ON-TARGETplus siRNA SDHD #07, targeting sequence: GCUUCGAACUCCAGUGGUC	Horizon Discovery	J-006305-07
ON-TARGETplus siRNA SDHD #08, targeting sequence: GCUUCCGGCUGCUUAUUUG	Horizon Discovery	J-006305-08
qRTPCR primer for human specific SDHC<br>Forward 5`-3`: ATGGGATCCGACACTTGATG<br>Reverse 5`-3`: GCTGGGAGCCTCCTTTCTT	This study	Sigma Aldrich
qRTPCR primer for human specific SDHD<br>Forward 5`-3`: CTTGCTCTGCGATGGACTATT<br>Reverse 5`-3`: AAGCCCAGGAGTTGGGTAAT	This study	Sigma Aldrich
qRTPCR primer for human specific CDH1<br>Forward 5`-3`: GATGGCGGCATTGTAGGT<br>Reverse 5`-3`: GCTCTGTCATGGAAGGTGCT	This study	Sigma Aldrich
qRTPCR primer for human specific VIM<br>Forward 5`-3`: TGAGATTGCCACCTACAGGAA<br>Reverse 5`-3`: GAGGGAGTGAATCCAGATTAGTTT	This study	Sigma Aldrich
qRTPCR primer for human specific TWIST1<br>Forward 5`-3`: GGCTCAGCTACGCCTTCTC<br>Reverse 5`-3`: CCTTCTCTGGAAACAATGACATCT	This study	Sigma Aldrich

qRTPCR primer for human specific ZEB1<br>Forward 5`-3`: GGAGGATGACACAGGAAAGG<br>Reverse 5`-3`: TCTGCATCTGACTCGCATTC	This study	Sigma Aldrich
qRTPCR primer for human specific S100A8<br>Forward 5`-3`: CAAGTCCGTGGGCATCAT<br>Reverse 5`-3`: GACGTCGATGATAGAGTTCAAGG	This study	Sigma Aldrich
qRTPCR primer for human specific S100A9<br>Forward 5`-3`: GTGCGAAAAGATCTGCAAAA<br>Reverse 5`-3`: CCAGCTGCTCTTGTCTGCATTT	This study	Sigma Aldrich
qRTPCR primer for RPL13A<br>Forward 5`-3`: AGCGGATGAACACCAACC<br>Reverse 5`-3`: TTTGTGGGGCAGCATACTC	This study	Sigma Aldrich
**SOFTWARE**		
Image Lab	Bio Rad	https://www.bio-rad.com/de-de/product/image-lab-software
VIS (Visiopharm Integrator sytem)	Visiopharm	https://visiopharm.com/
cellSens Dimension	Olympus	https://www.olympus-lifescience.com/de/software/cellsens/
GraphPad Prism8	GraphPad	https://www.graphpad.com/scientific-software/prism/
BD FACSDiva™ Software	BD Bioscience	https://www.bdbiosciences.com/en-us/instruments/research-instruments/research-software/flow-cytometry-acquisition/facsdiva-software
