# Supplementary material for: Th17 cells target the metabolic miR‐142‐5p–succinate dehydrogenase subunit C/D (SDHC/SDHD) axis, promoting invasiveness and progression of cervical cancers
Source: Mol Oncol. 2023 Nov 16;18(9):2157–78. doi: 10.1002/1878-0261.13546 (PMC11467798; doi:10.1002/1878-0261.13546)
Supplement: Supplementary file 9 — Table S2. Mutagenesis primers. [file MOL2-18-2157-s003.pdf]

Supplementary Table S2: Mutagenesis primers.

**Target gene**	**ID**	**mutagenesis primers name**	**Sequence** *(mutated sites are underlined)*
SDHC	NM_003001.5	5`-SDHC-mut<br>3`-SDHC-mut	GTATGTTCTTTTCCCTTTCGCGATTTCTCCTACCTTCC<br>GGAAGGTAGGAGAAATCGCGAAAGGGAAAAGAACATAC
SDHD	NM_003002.4	5`-SDHD-mut<br>3`-SDHD-mut	CTTAAAGAGAATCCATTCGCGATACGATTAGTATATG<br>CATATACTAATCGTATCGCGAATGGATTCTCTTTAAG
