# Supplementary material for: Th17 cells target the metabolic miR‐142‐5p–succinate dehydrogenase subunit C/D (SDHC/SDHD) axis, promoting invasiveness and progression of cervical cancers
Source: Mol Oncol. 2023 Nov 16;18(9):2157–78. doi: 10.1002/1878-0261.13546 (PMC11467798; doi:10.1002/1878-0261.13546)
Supplement: Supplementary file 10 — Table S3. Immunoreactive Score (IRS) according to Remmele & Stegner. [file MOL2-18-2157-s002.pdf]

Supplementary Table S3: Immunoreactive Score (IRS) according to Remmele & Stegner.

A (% of positive cells)	B (Staining intensity)	A x B= IRS
0 = no positive cells	0 = no positive cells	0-2 = negative
1 = <10% of positive cells	1 = weak color reaction	3-4 = weak
2 = 10-50% of positive cells	2 = moderate color reaction	6-8 = moderate
3 = 51-80% of positive cells	3 = strong color reaction	9-12 = strong
4 = >80% positive cells		
